# Single-molecule fluorescence multiplexing by multi-parameter spectroscopic detection of nanostructured FRET labels

**DOI:** 10.1038/s41565-024-01672-8

**Published:** 2024-05-15

**Authors:** Jiachong Chu, Ayesha Ejaz, Kyle M. Lin, Madeline R. Joseph, Aria E. Coraor, D. Allan Drummond, Allison H. Squires

**Affiliations:** 1 Pritzker School of Molecular Engineering, University of Chicago, Chicago, IL USA; 2 Department of Chemistry, University of Chicago, Chicago, IL USA; 3 Graduate Program in Biophysical Sciences, University of Chicago, Chicago, IL USA; 4 Interdisicplinary Scientist Training Program, Pritzker School of Medicine, University of Chicago, Chicago, IL USA; 5 Department of Biochemistry and Molecular Biophysics, University of Chicago, Chicago, IL USA; 6 Department of Medicine, Section of Genetic Medicine, University of Chicago, Chicago, IL USA; 7 Institute for Biophysical Dynamics, University of Chicago, Chicago, IL USA

**Keywords:** DNA nanotechnology, Techniques and instrumentation

## Abstract

Multiplexed, real-time fluorescence detection at the single-molecule level can reveal the stoichiometry, dynamics and interactions of multiple molecular species in mixtures and other complex samples. However, fluorescence-based sensing is typically limited to the detection of just 3–4 colours at a time due to low signal-to-noise ratio, high spectral overlap and the need to maintain the chemical compatibility of dyes. Here we engineered a palette of several dozen composite fluorescent labels, called FRETfluors, for multiplexed spectroscopic measurements at the single-molecule level. FRETfluors are compact nanostructures constructed from three chemical components (DNA, Cy3 and Cy5) with tunable spectroscopic properties due to variations in geometry, fluorophore attachment chemistry and DNA sequence. We demonstrate FRETfluor labelling and detection for low-concentration (<100 fM) mixtures of mRNA, dsDNA and proteins using an anti-Brownian electrokinetic trap. In addition to identifying the unique spectroscopic signature of each FRETfluor, this trap differentiates FRETfluors attached to a target from unbound FRETfluors, enabling wash-free sensing. Although usually considered an undesirable complication of fluorescence, here the inherent sensitivity of fluorophores to the local physicochemical environment provides a new design axis complementary to changing the FRET efficiency. As a result, the number of distinguishable FRETfluor labels can be combinatorically increased while chemical compatibility is maintained, expanding prospects for spectroscopic multiplexing at the single-molecule level using a minimal set of chemical building blocks.

## Abstract (web summary)

FRETfluors—nanostructures with Cy3 and Cy5 dyes and a DNA scaffold—are used to generate distinct spectroscopic signals from different configurations and mixtures of mRNA, dsDNA and proteins in an anti-Brownian electrokinetic trap for single-molecule multiplexed sensing.

## Main

Multiplexed measurements provide critical insights into the molecular compositions and interactions that govern complex nanoscale systems. Fluorescent labels offer sensitive, specific readout of molecular identity, enabling information-rich imaging and assays in a rainbow of colours for microscale objects. At the single-molecule level, colour-ratio-based multiplexing has been demonstrated by multiple groups for up to ~10 labels exhibiting unique colour combinations^1^ and up to 25 labels with a complex scheme of four-laser alternating excitation and four dyes^2^. However, low signal-to-noise ratios and overlapping spectra generally restrict single-molecule fluorescence multiplexing on the vast majority of microscopes to at most 3–4 colours, severely limiting opportunities for colour-ratio-based multiplexing at the single-molecule level.

To overcome this limitation, technologies for single-molecule fluorescence multiplexing utilize additional measurement dimensions to separate signals. Fixation and immobilization of samples enable barcode-based multiplexing strategies for up to thousands of labels by detecting unique combinations of molecular interactions across multiple rounds of readout for each molecule, providing patterning in temporal^3,4^, spatial^5–7^ or kinetic^8–10^ dimensions. But for living or dynamic samples where each molecule is measured only once, multiplexing must instead be encoded in spectroscopic information. Spectroscopic properties including fluorescence brightness and quantum yield, fluorescence lifetime, anisotropy and emission spectrum are routinely accessible with single-molecule sensing methods^11–14^. Spectroscopically multiplexed imaging at or approaching the single-molecule level has been demonstrated using up to nine labels^15^, and other sensing modalities have achieved up to six labels in complex sample mixtures with different fluorophores^1,16^. Although the use of chemically diverse fluorophores offers a potentially broad spectroscopic palette, further multiplexing is ultimately constrained by dissimilar chemical compatibility and labelling performance across different fluorophores.

One well-established means to vary spectroscopic signals using a limited number of chemical compounds is Förster resonance energy transfer (FRET) between fluorophores positioned on DNA nanostructures^17–19^. FRET is influenced by the photophysical properties and geometry of the donor and acceptor fluorophores. DNA nanotechnology can position and orient covalently linked fluorophores with sub-nanometre precision^5,20^, dictating spectroscopic properties^21–23^. Spectroscopic multiplexing using several DNA constructs^24^ and up to as many as 15 constructs^25^ has been demonstrated at the single-molecule level using simple DNA-FRET designs. However, practical limitations of DNA-FRET constructs including the spatial extent of FRET interactions (~10 nm); correlations and anticorrelations among spectroscopic variables; and tradeoffs among scaffold complexity, spectroscopic uniqueness, and error-free assembly and readout limit further multiplexing by DNA-FRET alone.

Separately, the photophysical properties of fluorophores on DNA can be influenced by local base sequence and attachment chemistries that alter the local physicochemical environment^26^. Cyanine dyes on DNA are a particularly well-studied class of constructs in this context^27^, and exhibit changing lifetimes and quantum yields^28–30^, orientation and base stacking^19,31,32^, system–bath coupling^33^, and torsion and isomerization^34–36^. These photophysical changes directly impact the energy transfer and fluorescence emission of DNA-FRET nanostructures^28,33^.

Here we have designed a set of DNA-FRET constructs for use as fluorescent labels in single-molecule multiplexing applications, called ‘FRETfluors’. FRETfluors are constructed from just three chemical building blocks (DNA, Cy3 and Cy5) and are designed for single-colour excitation. A combination of FRET, local DNA sequence and attachment chemistry tunes the spectroscopic emission of each FRETfluor. Here readout in an Anti-Brownian ELectrokinetic (ABEL) trap^37^ provides high-precision multi-parameter identification of labelled biomolecular targets at sub-picomolar concentrations including ssDNA, dsDNA, mRNA and proteins. Statistically optimal subsets of FRETfluors can be selected for multiplexing applications, demonstrated here for up to 27 FRETfluor labels.

## Results

### Design of FRETfluor labels

**Fig. 1:**
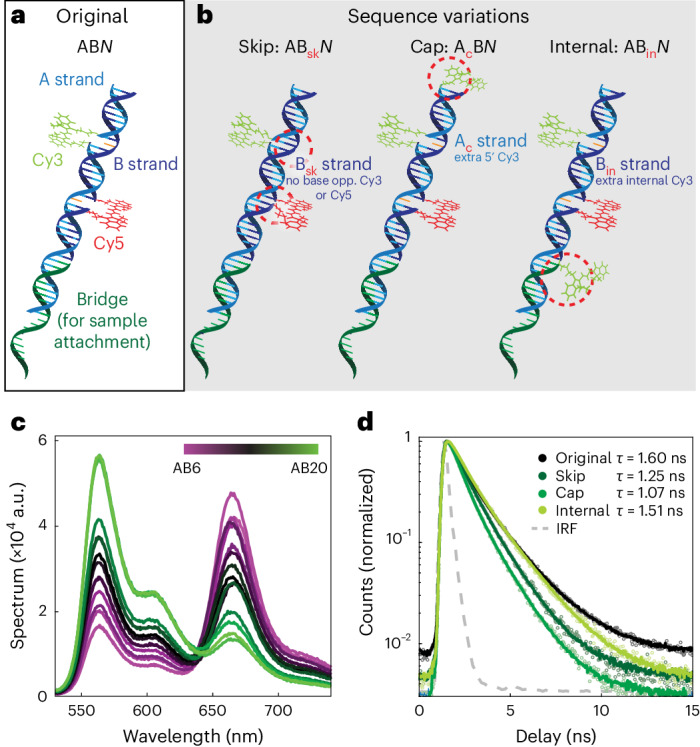
FRETfluor concept and design. **a**, FRETfluor design for AB*N* constructs (A strand, cyan; B strand, blue; unpaired bases, orange), shown here with a bridge (green) for the sequence-specific labelling of nucleic acids. **b**, FRETfluor design variations AB_sk_, A_c_B and AB_in_, used to create additional unique spectroscopic signatures. Key changes for each construct are highlighted with a dotted red circle. **c**, Bulk emission spectra of AB*N* constructs demonstrate that FRET tunes the emission, as expected. **d**, Fluorescence lifetime measurements of aggregated single-molecule data show that the Cy3 lifetime depends on the local DNA sequence and attachment chemistry (single-exponential fits are shown in solid lines; data, open circles; IRF, grey dotted line). Source data

To engineer a collection of fluorescent labels with unique spectroscopic signals, high chemical homogeneity and minimal structural complexity, we utilized a simple set of biomolecular building blocks: DNA oligomers functionalized with either Cy3 or Cy5 dye. We chose phosphoramidite incorporation of dyes into the DNA backbone to limit dipole rotational mobility^29^ and to improve photostability^38^. We first created a series of ‘AB*N*’ constructs incorporating non-sulfonated Cy3 and Cy5 into the ‘A’ and ‘B’ DNA strands, respectively, separated by *N* base pairs where 6 ≤ *N* ≤ 20 (Fig. 1a shows AB9). An additional ‘bridge’ strand enables the sequence-specific labelling of nucleic acid targets or the addition of functional groups for common labelling chemistries. FRETfluor constructs are similar in size to a fluorescent protein (~30 kDa) but with a higher aspect ratio. The single-exponential-fitted lifetime and background-subtracted brightness of the Cy3 donor in AB0 (lacking Cy5) were measured to be *τ*_AB0_ = 1.60 ± 0.03 ns and 0.31 ± 0.01 counts ms^–1^ μW^–1^, respectively. Supplementary Table 1 lists the FRETfluor oligo sequences.

We expected that the spectroscopic emission of different AB*N* FRETfluors would follow a smooth manifold in the detection parameter space (brightness, lifetime, emission spectrum or FRET efficiency), similar to previous FRET studies using DNA scaffolds^19,25^. By modifying the DNA sequence and attachment chemistry of the Cy3 donor or by including an additional Cy3, we created additional FRETfluor types with different photophysical properties from AB*N* (Fig. 1b). ‘Skip’ oligos, B_sk_, lack the unpaired bases opposing Cy3 and Cy5, lowering the lifetime and quantum efficiency of Cy3 compared with AB*N* constructs (*τ*_ABsk0_ = 1.25 ns ± 0.03; green brightness, 0.26 ± 0.01 counts ms^–1^ μW^–1^). ‘Cap’ oligos, A_c_, carry an additional single-tethered Cy3 at the 5’ end, increasing the brightness and lowering the net Cy3 lifetime (*τ*_AcB0_ = 1.07 ns ± 0.03; green brightness, 0.40 ± 0.01 counts ms^–1^ μW^–1^). ‘Internal’ oligos, B_in_, incorporate an additional Cy3 between the 3’ end of the bridge strand and the 5’ end of the B strand, increasing the brightness but only slightly affecting the net Cy3 lifetime (*τ*_ABin0_ = 1.51 ns ± 0.03; green brightness, 0.560 ± 0.015 counts ms^–1^ μW^–1^) (Supplementary Note 1, Supplementary Table 2 and Supplementary Fig. 1).

In bulk measurements, we observed the expected decrease in FRET efficiency for FRETfluors with increasing *N* (Fig. 1c). Single-exponent fits to the measured lifetime decays of Cy3 for each type of construct illustrate the effect of local sequence and attachment chemistry on donor lifetime (Fig. 1d). In total, 41 FRETfluor constructs were synthesized: 15 of AB*N*, 8 of AB_sk_*N*, 9 of A_c_B*N* and 9 of AB_in_*N*.

### Detection of FRETfluor labels in the ABEL trap

**Fig. 2:**
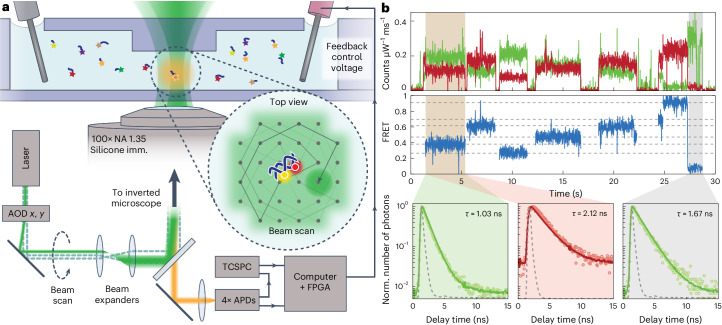
ABEL-trap-based detection of FRETfluors in a complex sample. **a**, Schematic of ABEL trap detection: FRETfluors (blue DNA; coloured stars) are detected in a microfluidic cell atop an inverted microscope. Here 532 nm pulsed laser excitation is scanned across the field of view using *x* and *y* acousto-optic deflectors (AODs). A FRETfluor in the trapping region fluoresces when it is co-localized at the scanned laser position, enabling closed-loop feedback control over its position via electrodes that apply *x* and *y* voltages to electrokinetically move the particle back to the trap centre. Spectroscopic data are simultaneously acquired (time-correlated single-photon counting (TCSPC); 4× APDs for polarization and red/green channels). FPGA, field-programmable gate array; NA, numerical aperture. **b**, Raw ABEL trap data showing signals from seven different FRETfluors over 30 s (10 ms binning). Background-subtracted brightness in the red and green channels increases during trapping events (top). FRET efficiency calculated from the red and green brightness traces (middle). The grey dotted lines indicate the expected FRET values for each class of FRETfluor. Fluorescence lifetime decays for the green and red channels during the first trapping event (brown background above; split into green and red backgrounds) versus donor lifetime when the acceptor is blinking or photobleached (grey background) (bottom). The IRF is shown by the grey dotted line. Source data

To spectroscopically characterize the single-molecule emission of each FRETfluor, we employed a custom-built ABEL trap (Fig. 2a). Originally developed by Cohen and Moerner^37^ to overcome common technical challenges in single-molecule measurements, the ABEL trap uses closed-loop feedback to electrophoretically counteract the effects of Brownian motion on single molecules in a solution-phase environment^39,40^. Critically, ABEL traps enable precise spectroscopic characterization of single molecules across multiple parameters, including brightness, fluorescence lifetime, anisotropy and emission spectrum^41–43^. With the record of applied voltages, the pattern of movement for a particle in the trap can be extrapolated and used to estimate hydrodynamic properties, including diffusion coefficient and electrophoretic mobility, allowing us to monitor the size of a FRETfluor or FRETfluor-labelled molecule^44^.

Raw ABEL trap data reveal different FRET values for FRETfluors in a mixture (Fig. 2b). Lifetime fitting confirms that the observed Cy3 lifetime is substantially shortened by FRET with Cy5 (Fig. 2b, bottom left) relative to the Cy3 lifetime after Cy5 photobleaches (Fig. 2b, bottom right). Supplementary Fig. 2 shows the annotated raw trapping data for a mixture of FRETfluors, with additional discussion in Supplementary Note 2.

Typical trapping throughput for FRETfluors in our ABEL trap setup is ~0.1 molecules s^–1^ pM^–1^; therefore, a measurement time of ~15 min is sufficient to analyse FRETfluor-labelled samples at concentrations down to tens of femtomolar (Supplementary Fig. 3 and Supplementary Note 3). The ABEL trap’s high sensitivity to ultralow concentrations of FRETfluors is a major advantage of our approach.

### Multi-parameter characterization of FRETfluor emission

**Fig. 3:**
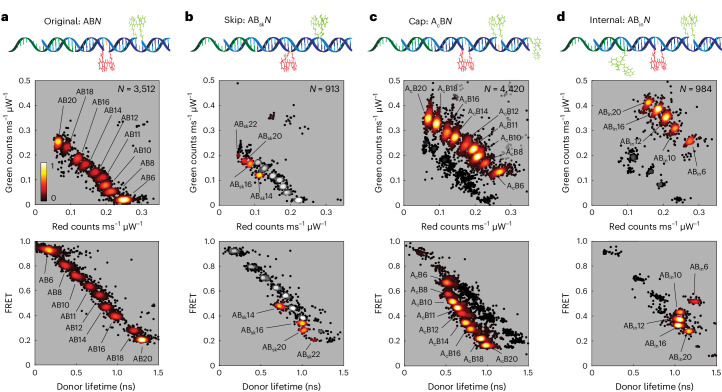
Tuning Cy3 photophysics shifts the spectroscopic properties of FRETfluor labels. The red–green and lifetime–FRET projections of the level-by-level data from trapped FRETfluor constructs show clusters in different regions of the measured multi-parameter space. Each point represents the average value of data from one level with duration of >150 ms; the number of levels passing this filter for each dataset is shown as *N* at the top-right corner of each red–green projection. The black–red–yellow heat map denotes the relative scatter-plot density from low (black) to high (yellow). **a**, Set of nine AB*N* constructs showing distinct clusters in both red–green (top) and lifetime–FRET projections (bottom). **b**, Data for four AB_sk_*N* constructs were taken along with nine AB*N* constructs (grey scale) to verify the shifted cluster locations. **c**,**d**, Data for nine A_c_B*N* constructs (**c**) and five AB_in_*N* constructs (**d**) similarly show distinct clusters that are distinguishable from the original AB*N* construct locations. Source data

The measured red and green channel brightness, donor lifetime and FRET efficiency data produce tight clusters for each type of FRETfluor construct (Fig. 3), indicating that these parameters can be used to classify single FRETfluors. Cluster size depends on the duration and photon content averaged into each data point; here we included trapping levels with durations of >150 ms. Under our experimental conditions, these levels average more than 5,000 photons each (Supplementary Fig. 4).

We tested FRETfluors individually and in various combinations to determine the characteristic emission properties and cluster widths for each of the 41 FRETfluor constructs (Supplementary Table 3). As expected, stepwise changes in donor–acceptor spacing within a FRETfluor construct type show correlated changes in the cluster position. For example, for a mixture of nine different AB*N* labels, red and green brightnesses are inversely correlated (Fig. 3a, top), and the donor lifetime is inversely correlated with the FRET efficiency (Fig. 3a, bottom).

Changes to the donor photophysics shift the FRETfluor clusters to other regions of the detection parameter space: four out of the eight AB_sk_*N* constructs, nine A_c_B*N* constructs and five AB_in_*N* constructs showed unique signals relative to the AB*N* constructs and one another (Fig. 3b–d). Constructs not included in Fig. 3 were determined to statistically overlap with one or more clusters beyond a 2.5% misclassification threshold (see the ‘Robust classification for mixtures of FRETfluors’ section) and therefore are not included here (Supplementary Figs. 5–7).

These results show that relatively small changes in dye photophysics, such as the 15–20% difference in donor brightness and lifetime between AB_sk_*N* and AB*N* FRETfluors, can produce uniquely identifiable spectroscopic signatures. We observed that donor brightness and lifetime changes were not perfectly correlated, confirming that both radiative and non-radiative lifetimes are influenced by the physicochemical environment^41^. Such tuning is useful for FRETfluor design; Supplementary Notes 4 and 5 and Supplementary Fig. 8 detail simulations showing that decoupled donor lifetime and brightness changes produce nearly orthogonal shifts in a FRET curve within the multi-parameter detection space.

To determine whether changing environmental conditions would substantially impact FRETfluor performance, we tested salt and pH across a physiological range (0 to 150 mM NaCl, pH 6.5 to 8.5). FRETfluor signals were consistent across all pH values tested and exhibited small (~10%) salt-induced reductions in the brightness of AB_in_*N*-type, AB*N*-type and A_c_B*N*-type constructs and in the donor lifetime of AB_in_*N*-type constructs (Supplementary Fig. 9 and Supplementary Note 6). These small shifts might be amenable to calibration such that FRETfluors could report on the local physicochemical environment with well-separated relative cluster locations so that each FRETfluor remains uniquely identifiable.

### Robust classification for mixtures of FRETfluors

**Fig. 4:**
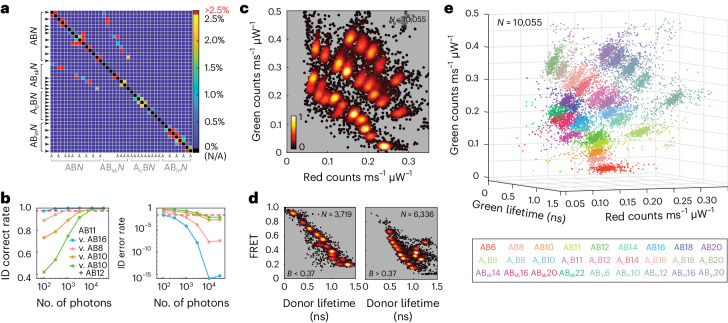
Selection and multiplexed detection of a near-orthogonal FRETfluor set. **a**, Misclassification probability calculated as a one-tailed Gaussian overlap for each pair of FRETfluors. True cluster identity (left) and incorrect cluster identity (bottom) intersect at squares coloured according to the probability of misidentification for each combination. The colour bar shows the probability of misclassification for each pair, capped here at 2.5% (red). Self-identification (black on-diagonal) is variable depending on the set of FRETfluors selected for use. **b**, Correct (left) and incorrect (right) identification rates for FRETfluor AB11 as a function of the number of photons used for analysis when considering confusion with either AB16 (blue), AB8 (pink), AB10 (orange) or both AB10 and AB12 (green). The cutoff of 97.5% is shown as a red dotted line. **c**, Red–green projection of data from multiplexed detection of 27 FRETfluors in a single sample. **d**, Two brightness slices of the same dataset shown in a lifetime–FRET projection, separated at *B* = 0.37 counts ms^–1^ μW^–1^. **e**, 3D projection of the same dataset coloured according to cluster membership for each of 27 FRETfluor labels per the key (bottom). Supplementary Video 1 shows a rotating view of this plot. In **c**–**e**, each point represents the average value of data from one level with duration of >150 ms; the number of levels passing this filter for each dataset is shown as *N* at top-right corner of each panel. Source data

In a mixture of FRETfluors, reliable classification of single molecules depends on experimental factors (such as measurement duration and precision) as well as on the set of FRETfluors used. We analysed all the possible pairwise combinations of our 41 FRETfluor constructs to determine which pairs presented higher chances of mutual misclassification. Each cluster of levels from the ABEL trap data was fit as a three-dimensional (3D) Gaussian distribution (red and green brightnesses and green lifetime), and one-tail integrations were performed over the parameter space to generate a confusion matrix (Fig. 4a). We set a 2.5% misclassification threshold for identifying unfavourable FRETfluor combinations. The maximum pairwise misidentification probability was found to be ~30% for AB_sk_10 and AB8 (Supplementary Fig. 10). Under 0.7% (33 of the 1,640 pairs) exceeded our 2.5% threshold, and the likelihood of misclassification for most pairs is vanishingly small (Supplementary Fig. 11). On the basis of this analysis, we identified a subset of 27 FRETfluors suitable for use in a single mixture, indicated by the arrows in Fig. 4a (bold typeface in Supplementary Table 3; Supplementary Fig. 12 shows the misclassification matrix).

In this work, we did not attempt to optimize the measurement throughput: FRETfluors were trapped until photobleaching or until their natural exit from the trap, usually after several seconds of measurement. To determine whether similar levels of discriminative power might be achieved with a higher throughput by limiting the trapping time, we examined the effect of the number of photons per point on the spread of the FRETfluor clusters (Supplementary Note 7). Here the typical total photon arrival rates are >25 kHz, and the levels used for analysis are typically based on several thousand photons. Limiting the number of photons per data point broadens the clusters and increases misclassification (Fig. 4b shows the case of a typical FRETfluor, AB11). For ~100 photons per point, AB11 can be correctly identified compared with its nearest neighbours, AB10 and AB12, with only about 45% (two-tailed) and 75% (one-tailed) accuracy. However, AB11 can still be readily differentiated from more distant clusters (AB8, 90.0% accuracy; AB16, 97.5% accuracy). At 3,000 photons per point, identification is at worst 92% (two-tailed nearest neighbours). Correct identification of AB11 from AB10 and AB12 surpasses 97.5% accuracy at ~6,000 photons per point. Thus, the throughput of FRETfluor identification could be improved by sampling each construct for long enough to gather ~10^4^ photons above the background, which requires only ~400 ms per trapping event (neglecting blinking effects).

We next tested our ability to experimentally distinguish this optimized FRETfluor set. We combined the complete set of 27 FRETfluors in a dilute sample mixture (~2 pM total; ~75 fM of each FRETfluor). Figure 4c shows a red–green projection illustrating clear separation of clusters; these clusters can be further differentiated in lifetime–FRET projections (Fig. 4d), here showing two brightness cuts above and below a brightness threshold of 0.37 counts ms^–1^ μW^–1^. A 3D view with each cluster coloured according to its most likely identity shows the distribution of cluster positions within the detection parameter space (Fig. 4e and Supplementary Video 1). The raw data in Supplementary Fig. 2 show this combination of FRETfluors and are annotated with tag identities. Cluster locations are consistent with the expected values (Supplementary Fig. 13).

### Wash-free labelling of biomolecular targets

The specificity of FRETfluors for biomolecular targets can be programmed using common attachment chemistries on the bridge strand for conjugation to, for example, antibodies, specific chemical linkers or nucleic acid sequences.

**Fig. 5:**
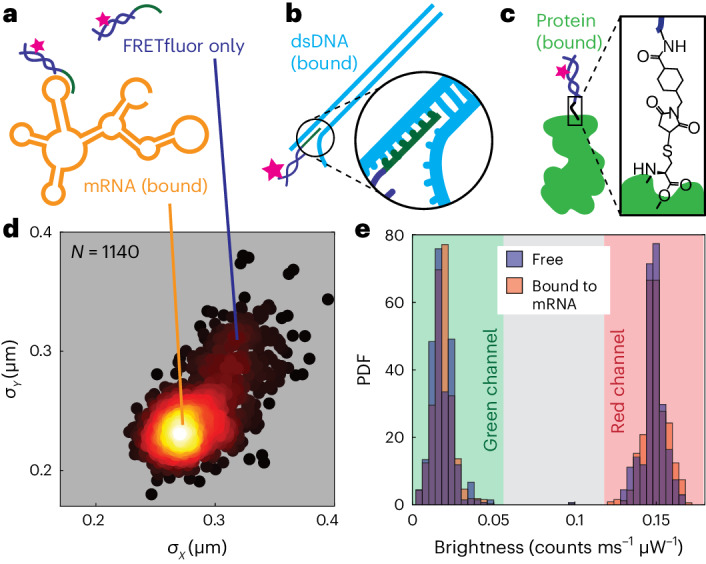
Sequence-specific labelling of mRNA, dsDNA and proteins by FRETfluors. **a**, FRETfluor tag (dark blue and pink) not bound to any target (top) and the same FRETfluor targeted to a loop on mRNA (bottom) via complementarity of the bridge strand (green). **b**, FRETfluor tag (dark blue and pink) targeted to dsDNA (cyan) via base complementarity of a bridge strand (green). **c**, Illustration of FRETfluor site-specifically labelling a protein (light green) via a maleimide–NHS ester bifunctional linker. **d**, Scatter plot of standard deviation of position in *x* and *y* for trapped molecules of AB6 FRETfluor targeted to *FLuc* mRNA. Points are coloured according to the local relative scatter-plot density; each point is calculated from localization trajectories based on 1,000 photons; *N* = 1,140 points are shown in the scatter plot. **e**, Normalized histograms showing the probability distribution functions of green (left) and red (right) signals for AB6 on *FLuc* mRNA are nearly identical for the free (blue histogram) and mRNA-bound (orange histogram) FRETfluor. Source data

We first targeted FRETfluors to specific nucleic acids via sequence complementarity between a bridge strand and RNA and DNA targets (Fig. 5a,b). Labelling specificity was confirmed by bulk electrophoretic mobility shift assay (EMSA) comparing FRETfluors equipped with either on- or off-target bridge sequences binding to an ssDNA target strand (Supplementary Fig. 14). We next tested RNA binding by designing bridge sequences complementary to regions predicted with high confidence to be part of a loop in the secondary structure of three mRNAs: enhanced green fluorescent protein mRNA (*EGFP*; 996 nt), firefly luciferase mRNA (*FLuc*; 1,929 nt) and ovalbumin mRNA (*OVA*; 1,438 nt)^45^. EMSA confirmed that off-target bridge sequences did not bind mRNA, whereas on-target sequences hybridized with each mRNA tested (Supplementary Note 8 and Supplementary Fig. 15). We similarly confirmed by EMSA that a FRETfluor with an on-target bridge sequence could invade and bind near the end of a dsDNA reverse transcription polymerase chain reaction (RT-PCR) product (Supplementary Fig. 16).

To site-specifically label proteins with FRETfluors, we utilized a bifunctional linker containing both an N-hydroxysuccinimide (NHS) ester group and a maleimide group, which we reacted with a primary amine on a FRETfluor and a cysteine sulfhydryl on the target protein (Fig. 5c). We verified covalent labelling by native polyacrylamide gel electrophoresis for two target proteins, poly-A binding protein (Pab1) and a Class A J-domain protein (Ydj1) from *Saccharomyces cerevisiae*, each mutated to have a single accessible cysteine (Supplementary Fig. 17).

Fluorescence labelling protocols usually require substantial washing or sample purification. In an ABEL trap, however, labelled biomolecules such as mRNA can be readily distinguished from free labels by estimating the transport properties of each trapped object. Objects with a larger hydrodynamic radius diffuse more slowly than free labels due to their size, leading to tighter confinement around the trap’s centre. We observed that the distribution of standard deviations in position from the trap centre in each direction, *σ*_*x*_ and *σ*_*y*_, for FRETfluors bound to a target mRNA exhibits two distinct clusters (Fig. 5d). We attribute the more tightly confined state to the correctly labelled FRETfluor–mRNA complex, whereas the less confined cluster is consistent with a trapped FRETfluor without the target present. These clusters can be used to separate labelled target molecules from free FRETfluors in post hoc analyses for wash-free labelling and detection of diverse biomolecular targets. In principle, sample throughput could also be increased by using this information on the fly to quickly reject free FRETfluors from the trap.

Cyanine dyes are well known to be sensitive to their environment in contexts beyond DNA attachment, for example, via protein-induced fluorescence enhancement^46,47^ or solution and local environment composition^27,34^. Here protein-induced fluorescence enhancement was neither observed on FRETfluor attachment to proteins nor were the FRETfluor signals altered by binding to mRNA or DNA (Fig. 5e). Together with the limited effects of salt and pH previously discussed, these results suggest that FRETfluor structure and attachment chemistry are dominant environmental influences on cyanine dye photophysics in this study, and that the FRETfluor structure may partially protect the cyanine dyes against interactions with the target molecules or solvent. Future use of non-isomerizing^48^ or otherwise photostabilized^49^ cyanine dyes could further protect FRETfluors from environmental effects.

### Detecting low-abundance targets in biomolecular mixtures

**Fig. 6:**
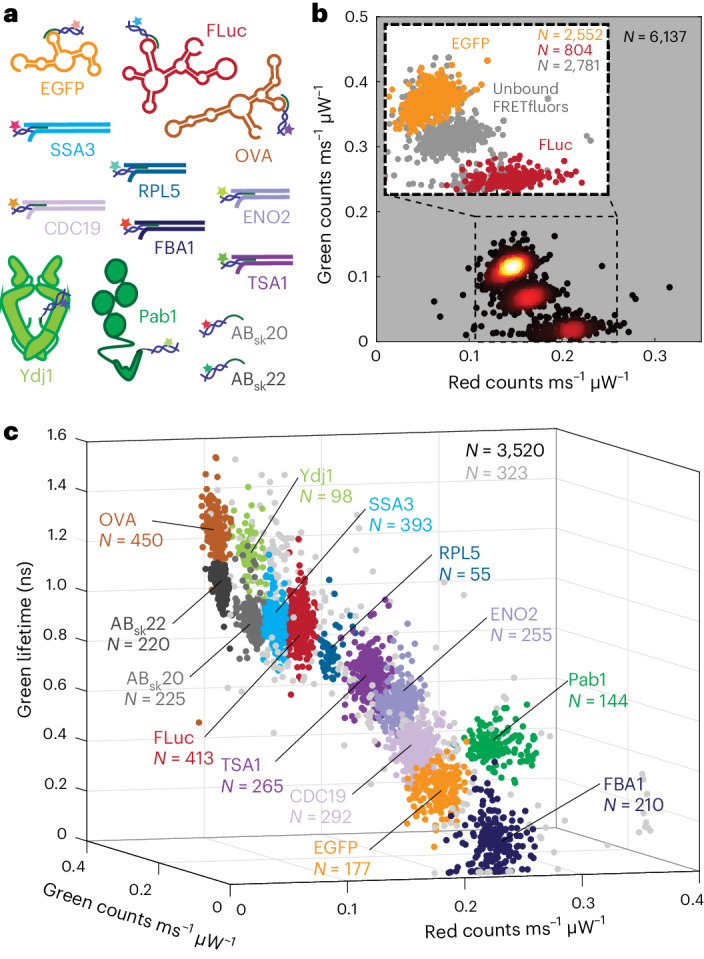
FRETfluor application to detect complex mixtures of biomolecules at low concentration. **a**, Identities of all mRNA (3), dsDNA (6), proteins (2) and target-less FRETfluor (2) samples. **b**, Scatter heat map of a red–green projection of data for a simple mixture of *EGFP* + AB12, *FLuc* + AB6 and AB10 (no target). Points are coloured according to the local relative scatter-plot density. The inset shows the scatter plot of signals from unbound FRETfluors of all types (grey) and bound *EGFP* + AB12 (orange) and *FLuc* + AB6 (red). Each point in the main panel and the inset represents 1,000 photons, with ‘bound’ and ‘unbound’ assignments based on localization trajectories and red–green brightness taken from the corresponding level with duration of >100 ms; *N* = 6,137 total points. **c**, 3D projection of spectroscopic data for a complex mixture of FRETfluor-labelled mRNA, dsDNA and proteins (*N* = 3,520 points). Cluster colours are assigned as per **a** (*N* indicated for each cluster in the corresponding colour), with unassigned data points shown in light grey (*N* = 323 points). Each point represents the average value of data from one level with duration of >150 ms. Supplementary Fig. 18 provides the red–green and lifetime–FRET projections of this dataset. Source data

To explore the suitability of FRETfluors for multiplexed detection of low-abundance biomarkers, we tested both simple and complex mixtures of FRETfluor-labelled mRNA, dsDNA and proteins (Fig. 6a). We first performed wash-free labelling and readout of a 1:1 mixture of *FLuc* and *EGFP* mRNAs labelled with FRETfluors AB6 and AB12, respectively. We also included an off-target-free FRETfluor, AB10, to control non-specific labelling. We observed three distinct spectroscopic populations corresponding to AB6, AB10 and AB12 (Fig. 6b). Separating bound and unbound populations in each cluster revealed that both AB6 and AB12 bind to their target mRNA with 71% and 73% binding efficiency, respectively, whereas all AB12 was free, confirming that off-target labelling or cross-reactivity did not occur and that FRETfluor spectroscopic signatures are unchanged by target binding (Fig. 6b (inset) and Supplementary Fig. 18).

Finally, we tested multiplexed detection of low-abundance biomarkers in a more complex mixture, as might be found in biomedical or environmental samples. We separately labelled mRNAs (*EGFP*, *FLuc* and *OVA*), proteins (Pab1 and Ydj1) and dsDNA fragments produced by RT-PCR from abundantly expressed and stress-response-related transcripts in *S. cerevisiae*^50^ (*FBA1*, *CDC19*, *ENO2*, *TSA1*, *RPL5* and *SSA3*), with a subset of the 27-FRETfluor combination tested above (Supplementary Tables 4 and 5). Together with two off-target FRETfluors as controls, the analysis of a low-concentration mixture of all the labelled targets (~350 fM each) shows all the FRETfluors present with their expected spectroscopic signatures (Fig. 6c); clusters do not shift on binding the targets. A closer examination of each cluster (Supplementary Fig. 19) shows that bound and unbound FRETfluors can be differentiated as expected, and that FRETfluors do not cross-react to label incorrect targets.

We found that cluster occupancy was approximately—but not exactly—reflective of the mixture stoichiometry. Discrepancies may arise from complicating factors such as trapping bias or dye photophysics, which could influence both number and proportion of the observed levels meeting the analysis filter criteria. We expect that these effects could, in principle, be calibrated out for each sample and label combination by comparison with known standards.

## Conclusion

Our FRETfluor designs utilize site-specific tunability of dye photophysics as an additional multiplexing dimension to complement—and combinatorially expand—the multiplexing power of FRET with a minimal set of chemical building blocks. The approach demonstrated here is, in principle, cross-compatible with strategies for single-molecule spectroscopic multiplexing that have been proposed by others, including additional excitation lasers, dye ratios or colours^1,2,25^; orientational control of dyes to influence polarization^19,33^, making use of the full distributions available across the multiple detected parameters^15^ rather than simplified or averaged values as we show here; and barcoding-type multiplexing strategies such as DNA-PAINT^4,8^. Other molecular scaffolds or fluorophore types could be used to create FRETfluors, and scaffold geometries and sizes could be altered and optimized. Even simple changes, such as site-specific tuning of acceptor properties, would further expand the number of identifiable constructs.

Our results here provide a proof of concept for the use of dozens of FRETfluors in applications that require characterization of dilute, highly heterogeneous mixtures of different types of biomolecules. Further development of FRETfluor sensing platforms could parallelize sample readout and incorporate real-time identification for a higher throughput. We anticipate that application-specific optimization will be necessary to balance tradeoffs between throughput and labelling efficiency of biomarkers (Supplementary Note 9 provides an additional discussion). In principle, FRETfluors could also be used in a wide-field imaging format, given sufficiently sensitive wide-field fluorescence lifetime imaging microscopy capabilities, although a wide-field approach might struggle to capture the size information necessary for wash-free detection. Finally, our present analysis and classification are based on average values extracted from discrete brightness levels, but we expect that FRETfluor data would lend itself to more sophisticated analyses involving machine learning, which could take full advantage of the information-rich, time-dependent data for higher-fidelity classification of FRETfluor identity and target-binding status.

## Methods

### DNA oligo samples and FRETfluor preparation

All oligos were purchased with fluorophores from IDT and purified by high-performance liquid chromatography. Supplementary Table 1 and Supplementary Data 1 provide full sequences for all oligos. Most oligos include either iCy3 or iCy5 internal modifications (Cy3 and Cy5 are non-sulfonated). The labelling efficiencies of each strand were 70–90%, as obtained by absorption measurements. The dsDNA constructs were annealed by mixing the complementary strands at 5 µM concentration in TE buffer (pH 8.0), heating to 90 °C for 2 min and slowly cooled down to 25 °C with steps of 0.5 °C per 20 s. DNA samples were stored at 4 °C before use. Bulk fluorescence emission was characterized with Fluorolog-3 with FluorEssence in shared facilities at the University of Chicago. Bulk fluorescence lifetime measurements for Cy3 were taken with a ChronosBH instrument with magic angle detection and confirmed via bulk measurements on the ABEL setup. For bulk characterization, the samples were excited with a 520 nm laser to minimize direct excitation of the acceptor.

### Single-molecule characterization in the ABEL trap

A custom ABEL trap was constructed as previously described^43,44,51^, incorporating excitation with a pulsed (60 MHz) supercontinuum laser (Leukos ROCK 400-4) paired with an acousto-optic tunable filter (Leukos TANGO VIS) to output 532 nm excitation light. The excitation spot was steered in the sample plane using two acousto-optic deflectors (MT110-B50A1.5-VIS) arranged orthogonally and driven by a direct digital synthesizer (DDSPA2X-D8b15b-34). Fluorescence photons are split into red and green channels using a dichroic filter (Chroma T610lpxr). Each channel is then split into *s*- and *p*-polarized light using a polarizing beamsplitter (Thorlabs) and focused onto separate avalanche photodiodes (APDs; Excelitas SPCM-AQRH-14-ND; a total of four). For each detected photon, a time-correlated single-photon counter (PicoQuant MultiHarp 150) records the time as well as the colour and polarization channel in which it arrived.

APD signals are also sent to a field-programmable gate array (NI PCIe-78656) that controls the ABEL trap. On the basis of the position of the laser on the arrival of each fluorescent photon, taking into account a pre-calibrated lag, the position of the fluorescent molecule is estimated via a noise-rejecting Kalman filter^44^. XY voltages proportional to the estimated displacement of the trapped particle relative to the centre of the trap are passed to a 10× voltage amplifier (Pendulum F10AD). The amplified voltages are applied to a quartz microfluidic sample cell via platinum electrodes that sit in four reservoirs at the cardinal points of the microfluidic cell.

The microfluidic cells are cleaned and passivated before each trapping experiment. Microfluidic cells were cleaned in a piranha solution (3:1 mixture of sulfuric acid and hydrogen peroxide) overnight and then extensively rinsed with ultrapure deionized water. The microfluidic cells were incubated in 1 M KOH for 10 min and then passivated using mPEG-silane (Laysan Bio MPEG-SIL-5000, 50 mg ml^–1^) in 95% ethanol, 5% water with 10 mM acetic acid for 48 h (ref. ^25^). The microfluidic cells were rinsed with ultrapure deionized water and incubated with 1% Tween^52^ for 10 min and rinsed again thoroughly before adding the sample for measurements.

Immediately before measurement, FRETfluors were diluted to a total concentration between 1 and 5 pM in a buffer containing 20 mM HEPES (pH 7.4), 3 mM Trolox and an oxygen-scavenging system (~60 nM protocatechuate-3,4-dioxygenase and 2.6 mM protocatechuic acid).

### Analysis of ABEL trap data

All data analysis was performed in MATLAB^43^. Briefly, the photon arrival times recorded by TCSPC were used to construct a 10-ms-binned time trace, for which the background levels were identified for each channel using an Aikake-information-criteria-optimized *K*-means clustering algorithm. The raw photon arrival timestamps were used to identify brightness change points in each channel using an established maximum likelihood algorithm^53^, which were merged into a single list of change points. Data between each pair of change points were assigned to one ‘level’.

For each level, the background-subtracted brightness in all four detection channels was determined and used to calculate a FRET value. Note that here we use both FRET and donor lifetime parameters only for the purpose of separating distinct spectroscopic signals; therefore, further corrections are not necessary for the purpose of this analysis. To facilitate comparison of our data with the data generated by other labs, we include the FRET correction parameters for our setup (Supplementary Note 10). For the donor lifetimes, we only used photons from the green parallel channel, fitted by maximum likelihood using iterative convolution with the instrument response function (IRF) to a single-exponential decay for each level, as previously described^14,43,54,55^. IRFs were collected using a short-lifetime fluorescent dye (malachite green). Although double- and triple-exponential decays provide better fits to the single-molecule lifetime data, for the purpose of this work and given the low photon count in many levels, we observed more robust label classification using single-exponential fits for the lifetime data. Data from levels of duration greater than 150 ms were used for subsequent analysis and can be viewed as individual points in the figure scatter plots.

For diffusion-based analyses, levels were further binned into groups of *M* photons (statistical separation of clusters based on *M* photons, calculation of *x*–*y* position fluctuations within the trap); photons at the end of each level were unused. In these cases, each point on the scatter plot represents data from one group of *M* photons.

### Statistics and reproducibility

Data collection duration and sample concentration (sample size) were designed to generate thousands of trapping events per experiment (Supplementary Fig. 3 and Supplementary Note 3). Data levels shorter than 150 ms were generally excluded from further analysis, as described above, because we found that the inclusion of shorter levels reduced the FRETfluor classification accuracy. For the simple mRNA mixture, a threshold of 100 ms was used. Experiments were not randomized. Investigators were not blinded to allocation during experiments and outcome assessment.

To determine FRETfluor identity, *K*-means clustering in three dimensions (red and green brightness and donor lifetime) was used for the initial classification of events from each dataset involving more than one FRETfluor label. The mean and standard deviation in each cluster was determined using a 3D Gaussian fit without covariance to the cluster after rejecting all the outliers (>3*σ* from the initial cluster mean). Means and standard deviations from the same FRETfluors across different datasets collected on different days were compared to verify the consistency of cluster sizes and locations.

Statistical pairwise overlap of these normalized 3D Gaussians (green brightness, red brightness and green lifetime) was used to calculate the probability of pairwise misclassification, defined as the summed probability in the overlapping tails of each pair of distributions.

### Nucleic acid labelling

Supplementary Table 1 and Supplementary Data 1 give the bridge strand sequences (BR) for targeting all ssDNA, mRNA and dsDNA, including null (non-targeting) sequences, as well as the amine-modified bridge sequence for site-specific protein labelling. Bridge sequences were selected to target loops in the predicted mRNA structures^45^ or the ends of dsDNA structures. FRETfluor constructs were incubated with the ssDNA target during the annealing process, with binding confirmed by EMSA. Polyacrylamide gels (12%) for ssDNA binding were run at 200 V in Tris-borate-EDTA buffer (TBE).

We used mRNA for *EGFP*, *OVA* and *FLuc*, all of which were commercially available (Trilink BioTechnologies, CleanCap mRNA nos. L-8101, L-7610 and L-8102, respectively) (Supplementary Note 8 and Supplementary Data 1). Incubation with the mRNA samples was carried out at 37 °C for 18 h in a labelling buffer (TE, pH 8.0) containing RNase inhibitor (10 *U* μl^–1^, SUPERase•In RNase Inhibitor). After annealing, both EMSA and ABEL trap data confirmed mRNA binding. EMSA for mRNA was performed at room temperature on agarose gels (3%) run at 110 V in 0.5× Tris-acetate-EDTA buffer (TAE). The maximum current was set to 50 mA.

To generate the dsDNA targets for FRETfluor binding, total RNA was extracted from wild-type BY4742 yeast (*S. cerevisiae*) grown in a yeast extract peptone dextrose medium to an optical density of 0.3–0.4, using a Direct-zol RNA purification kit (Zymo Research) with on-column DNase I treatment for at least 15 min. The purified RNA was reverse transcribed (iScript Select, Bio-Rad) with custom gene-specific primers (IDT; sequences given in Supplementary Table 5 and Supplementary Data 1). The targeted genes were *FBA1*, *CDC19*, *ENO2*, *TSA1*, *RPL5* and *SSA3*. The combined RNA and cDNA was further amplified by conventional PCR (Q5 Hot Start Master Mix, New England Biolabs) using asymmetric priming to favour production of the coding DNA strand (1 µM forward primer, 25 nM reverse primer). The final target DNA amplicons ranged from 516 to 1,262 bases in length. The RT-PCR products were then hybridized with the designed bridge by heating to 90.0 °C for 2 min and slowly cooling down to 25.0 °C with steps of 0.5 °C per 20 s. Targeting bridges were designed to attach to the 25 bases next to the forward-primer region of the target sequence to avoid competing against the free primer. For target labelling, FRETfluor tags hybridized to the appropriate bridge strand were introduced and the mixture was incubated at room temperature for 3 h. Here 3% agarose gels were run at 110 V in 1× TAE, and the target bands were cut and soaked in TE buffer (pH 8.0) overnight for extraction. After a clarifying spin (10,000*g*, 2 min), 150 μl of the supernatant was removed as the final dsDNA FRETfluor stock sample.

### Protein labelling

FRETfluors were conjugated to proteins via a bifunctional small-molecule linker (Pierce SMCC, No-Weigh Format, Thermo Fisher) containing both an NHS ester group and a maleimide group. FRETfluors were pre-hybridized with a bridge DNA strand modified at the 5’ end with a primary amine (BR_amine_). The assembled FRETfluor was reacted with a linker at an approximately 500:1 molar ratio (linker to FRETfluor) in phosphate-buffered saline at pH 7.4 (Gibco) for 5 h at room temperature, covered and shaken at 300 r.p.m. The FRETfluor–linker mixture was then buffer exchanged into fresh buffer to separate unreacted linker from FRETfluor (Zeba 7K MWCO, Thermo Fisher).

In parallel, the target proteins mutated to have a single accessible cysteine (*S. cerevisiae* Pab1 C70A/C119A/C368A/A577C and Ydj1 C29A/C370F) were reduced in 5 mM tris(2-carboxyethyl)phosphine for 45 min at room temperature and then buffer exchanged into reducing-agent-free phosphate-buffered saline at pH 7.4 (Gibco) by spin column (Zeba 7K MWCO, Thermo Fisher). Protein concentration after buffer exchange was measured by absorbance at 280 nm (NanoDrop One).

The reduced protein and FRETfluor–linker samples were then combined in an approximately 100:1 molar ratio (protein monomer:FRETfluor) and incubated for 2 h at room temperature, covered and shaken at 300 r.p.m.

The protein–linker–FRETfluor mixture was mixed with 50% v/v glycerol to a final concentration of 10% glycerol and loaded onto a polyacrylamide gel (Mini-PROTEAN TGX 4–20%, Bio-Rad). The samples were electrophoresed at 200 V for 20 min in a Tris/glycine/sodium dodecyl sulfate running buffer. Two reference lanes (Pab1 and Ydj1) were excised and stained with Coomassie (Gel Code Blue, Thermo Fisher) to visualize the protein. The gel was pieced back together and imaged (ChemiDoc, Bio-Rad) in Cy5 and Cy3 fluorescence channels to determine the location of FRETfluors on the gel. Gel fragments (unstained) containing protein and bound FRETfluor, but not free FRETfluor, were excised and incubated in the dark at 30 °C under shaking for 10 h with 500 μl gel extraction buffer (50.0 mM Tris–HCl, 150.0 mM NaCl, 0.1 mM EDTA, pH 7.5). After 10 h, the buffer was separated from the gel pieces and subjected to a clarifying spin (10,000*g*, 10 min). Then, 300 μl of the supernatant was removed as the final protein FRETfluor stock sample and stored at 4 °C.

### Sample preparation for mixture applications

After individually preparing all the FRETfluor-labelled mRNA, dsDNA and protein samples, fluorescence correlation spectroscopy was used to estimate each sample concentration. The ABEL trap setup (beam not scanned, feedback off) was used to collect these data. The samples were diluted to 50–500 fM each to generate a concentration of ~5 pM total sample in a buffer containing 20.0 mM HEPES (pH 7.4), 3.0 mM Trolox and an oxygen-scavenging system (~60 nM protocatechuate-3,4-dioxygenase and 2.6 mM protocatechuic acid).

## Online content

Any methods, additional references, Nature Portfolio reporting summaries, source data, extended data, supplementary information, acknowledgements, peer review information; details of author contributions and competing interests; and statements of data and code availability are available at 10.1038/s41565-024-01672-8.

### Supplementary information

Supplementary Information Supplementary Figs. 1–24, Tables 1–5 and Notes 1–10.

Supplementary Data 1 DNA oligo, primer and mRNA sequences.

Supplementary Video 1 Rotating view of the data in Fig. 4e.

### Source data

Source Data Fig. 1 Statistical source data for Fig. 1c,d.

Source Data Fig. 2 Statistical source data for Fig. 2b.

Source Data Fig. 3 Statistical source data for Fig. 3a–d.

Source Data Fig. 4 Statistical source data for Fig. 4a–e.

Source Data Fig. 5 Statistical source data for Fig. 5d,e.

Source Data Fig. 6 Statistical source data for Fig. 6b,c.

## Data Availability

The data supporting the findings of this study are available within this Article and its Supplementary Information. Source data are provided with this paper and are available via Zenodo at 10.5281/zenodo.10895539 (ref. ^56^). Oligo sequences for all the unique materials created in this work are detailed in Supplementary Table 1 and Supplementary Table 5 and are commercially available. Source data are provided with this paper.
