# Supplementary material for: Single-molecule fluorescence multiplexing by multi-parameter spectroscopic detection of nanostructured FRET labels
Source: Nat Nanotechnol. 2024 May 15;19(8):1150–7. doi: 10.1038/s41565-024-01672-8 (PMC11329371; doi:10.1038/s41565-024-01672-8)
Supplement: Supplementary file 1 — Supplementary Figs. 1–24, Tables 1–5 and Notes 1–10. [file 41565_2024_1672_MOESM1_ESM.pdf]

nature nanotechnology

Article

https://doi.org/10.1038/s41565-024-01672-8

Single-molecule fluorescence multiplexing by multi-parameter spectroscopic detection of nanostructured FRET labels

In the format provided by the authors and unedited

SUPPLEMENTARY INFORMATION:
Single-molecule fluorescence multiplexing by multi-parameter spectroscopic detection of nanostructured FRET labels

Jiachong Chu*, Ayesha Ejaz*, Kyle M. Lin, Madeline R. Joseph, Aria E. Coraor, D. Allan Drummond, and Allison H. Squires

Table of Contents

Table of Contents 1

Supplementary Figures 3

Supplementary Figure 1: Single-molecule brightness and lifetime data for donor-only constructs 3

Supplementary Figure 2: Extended raw data trace for ABEL trapping of single tags. 4

Supplementary Figure 3: Limit of detection for FRETfluors in the ABEL trap 5

Supplementary Figure 4: Photons per data level. 6

Supplementary Figure 5: Additional constructs from the ABN series 7

Supplementary Figure 6: Additional constructs from the $AB_{sk}N$ series. 8

Supplementary Figure 7: Additional constructs from the $AB_{in}N$ series. 9

Supplementary Figure 8: Simple geometrical model for FRET on DNA 10

Supplementary Figure 9: FRETfluors in different salt and pH conditions 11

Supplementary Figure 10: Detail of the pairwise distribution analysis 12

Supplementary Figure 11: Pairwise misidentification distributions 13

Supplementary Figure 12: Statistical selection of a near-orthogonal FRETfluor set 14

Supplementary Figure 13: Cluster locations for 27-tag FRETfluor set 15

Supplementary Figure 14: Binding to ssDNA 16

Supplementary Figure 15: FRETfluor binding to mRNA 17

Supplementary Figure 16: FRETfluor binding to dsDNA 18

Supplementary Figure 17: FRETfluor binding to proteins. 19

Supplementary Figure 18: FRETfluor labeling and readout of an mRNA mixture 20

Supplementary Figure 19: FRETfluor labeling and readout of mRNA, dsDNA, and protein mixture 21

Supplementary Figure 20: Full gel scan from Supplementary Fig 14b. 22

Supplementary Figure 21: Full gel scan of Supplementary Fig. 15b. 23

Supplementary Figure 22: Full gel scan of Supplementary Fig 15c. 24

Supplementary Figure 23: Full gel scan of Supplementary Fig 16b. 25

Supplementary Figure 24: Full gel scan of Supplementary Fig 17 26
Supplementary Tables 27
Supplementary Table 1: DNA oligomer sequences for FRETfluors and targeting bridges 27
Supplementary Table 2: Lifetime fitting of donor-only constructs 29
Supplementary Table 3: FRETfluor cluster data 30
Supplementary Table 4: Heterogeneous mixture components and labels 32
Supplementary Table 5: Target genes and RT-PCR primers 33
Supplementary Notes 34
Supplementary Note 1: Sequence- and attachment-dependent photophysics of Cy3 34
Supplementary Note 2: Raw trapping events 36
Supplementary Note 3: Trapping event rate and limit of detection 37
Supplementary Note 4: Simulation of energy transfer between Cy3 and Cy5 on dsDNA 38
Supplementary Note 5: Effect of changing donor photophysical properties on FRET 40
Supplementary Note 6: Effects of salt and pH on FRETfluor properties 41
Supplementary Note 7: Identification of FRETfluors depends on # photons available 42
Supplementary Note 8: Sequence of mRNAs and FRETfluor binding sites 43
Supplementary Note 9: Labeling efficiency and measurement throughput tradeoffs 45
Supplementary Note 10: Instrument correction parameters 46
Supplementary References 48

Supplementary Figures

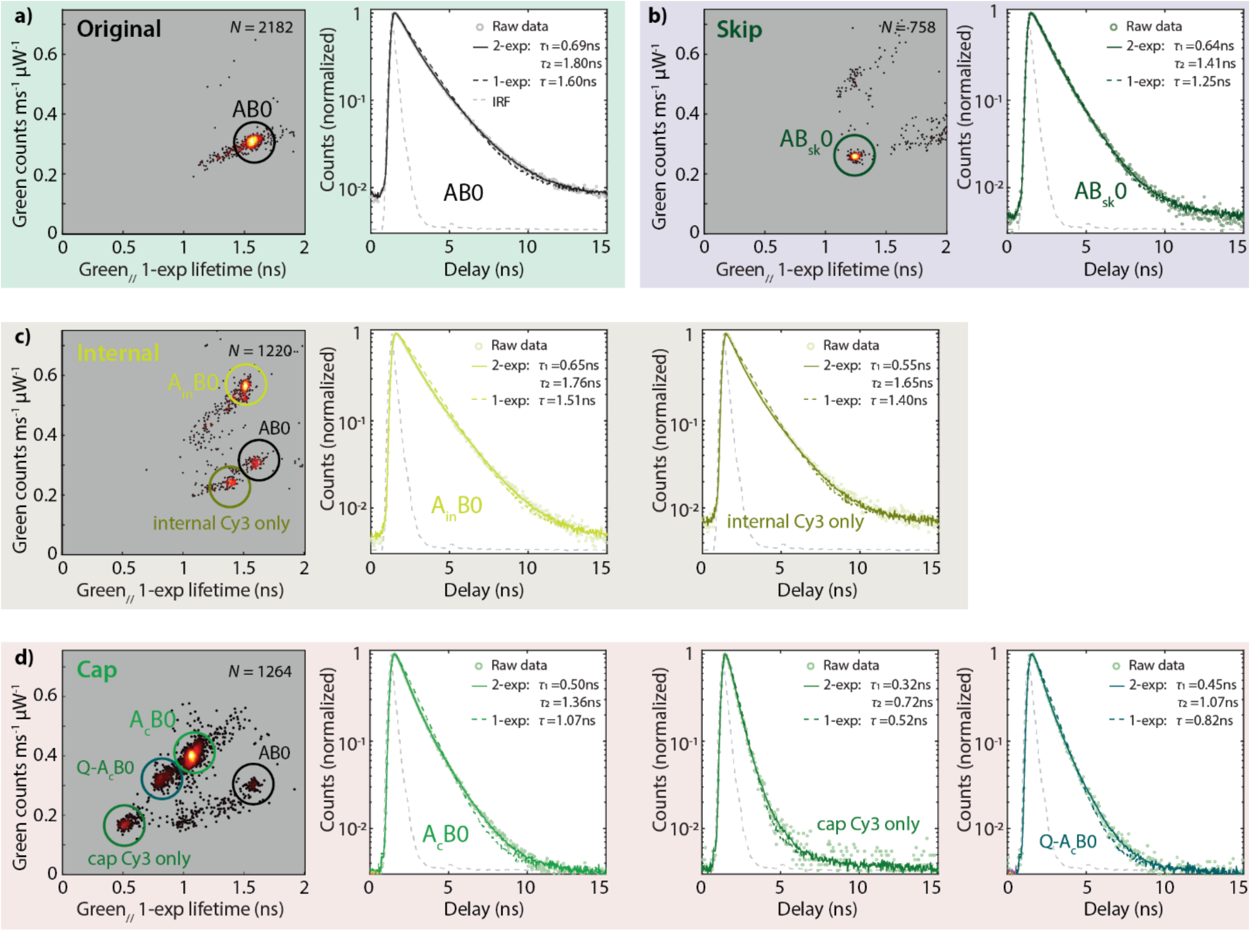

Supplementary Figure 1: Single-molecule brightness and lifetime data for donor-only constructs.
a) Levels for construct AB0 show one cluster. b) Levels for construct $AB_{sk}0$ also show just one population with a slightly shorter lifetime and dimmer brightness. Note that the changes in brightness and lifetime are not proportional to one another. c) Levels for construct $A_{in}B0$ show three populations: the original AB0 population, a population for the internal Cy3 label, and the majority population when both Cy3s are on. d) The levels for A_cB0 constructs show multiple states, of which the brightest population has both Cy3s on (as verified by allowed transitions into and out of this state). The AB0 population is unchanged. The cap-Cy3-only population can be deduced to be the dim, short-lifetime population. The fourth population is unknown. In all cases, the 2-component lifetime fits are superior, but the 1-component lifetime fits adequately represent the weighted average of the better fits. IRFs are shown as gray dashed curves.

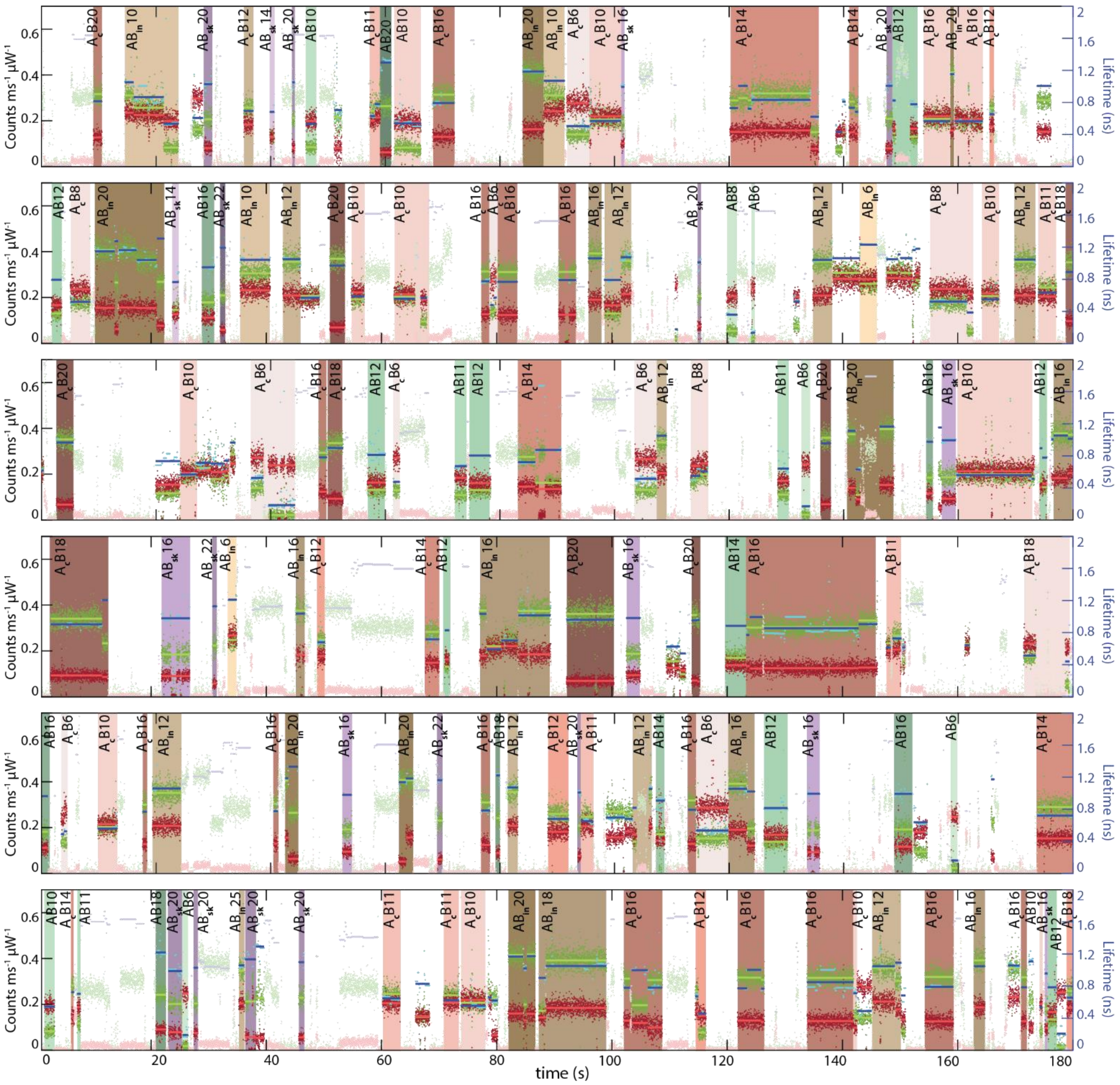

Supplementary Figure 2: Extended raw data trace for ABEL trapping of single tags. Raw trapping data is shown for green brightness, red brightness, and green channel lifetime for 27 unique FRETfluor tags over a representative 18-minute data set. After events were filtered out when the red channel brightness is lower than 0.5 counts ms^{-1} μW^{-1}, the rest of the events were identified according to the most likely cluster identity from Supplementary Table 3, with a limit of 1 standard deviation from the mean. Most unclassified (dim) events are excluded because they are donor-only. Highlight colors and FRETfluor labels correspond to the populations illustrated in Supplementary Fig. 13 and detailed in Supplementary Table 3. Desaturated data indicates a donor-only event; saturated levels without colorblocks indicate an event that could not be classified.

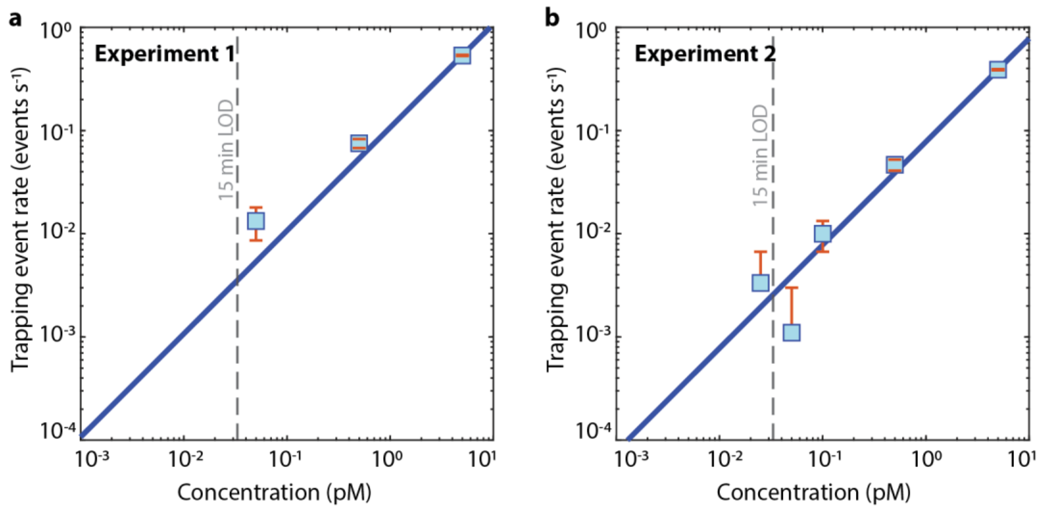

Supplementary Figure 3: Limit of detection for FRETfluors in the ABEL trap. a) Observed trapping event rate for AB12 at increasing concentration (50 fM, 1 pM, 5 pM; 3 data sets of 5 min each; fit slope 0.08 events s^{-1} pM^{-1}). b) Observed trapping event rate for AB12 at decreasing concentrations (5 pM, 500 fM, 100 fM, 50 fM, 25 fM; 3 data sets of 5 min each; fit slope 0.1 events s^{-1} pM^{-1}). Gray dotted lines indicate the calculated limit of detection (LOD) for a 15-minute measurement, ~33 fM (see Supplementary Note 3 for details).

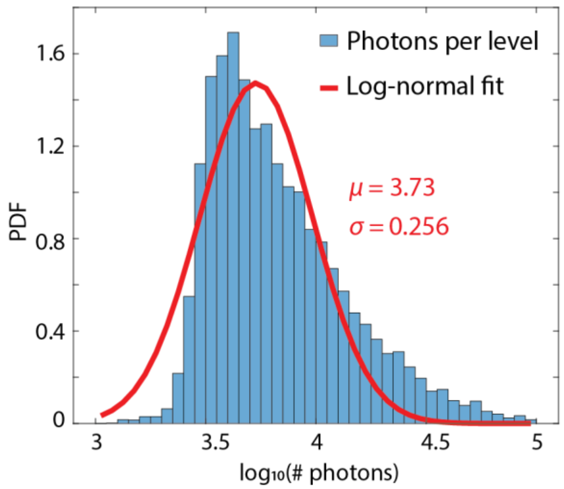

Supplementary Figure 4: Photons per data level. Log-normal histogram and fit of the number of photons per measured level for a mixture of FRETfluors (red and green channels combined, background subtracted). Mean of fit: $10^{3.73}$ = 5354 photons (-σ: 2971 phot, +σ: 9650 phot).

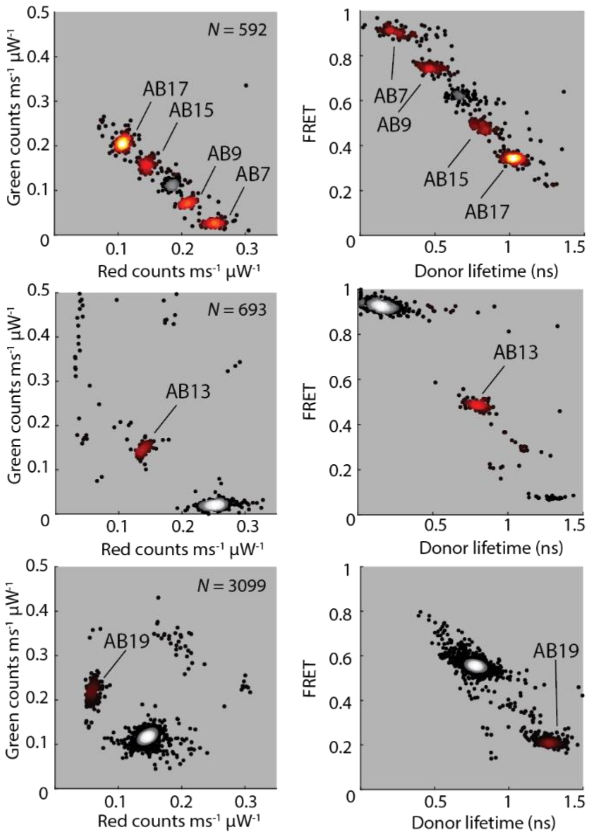

Supplementary Figure 5: Additional constructs from the AB*N* series. Red-green and lifetime-FRET projections of data from trapped FRETfluors with design variations show clusters in different regions of the parameter space.

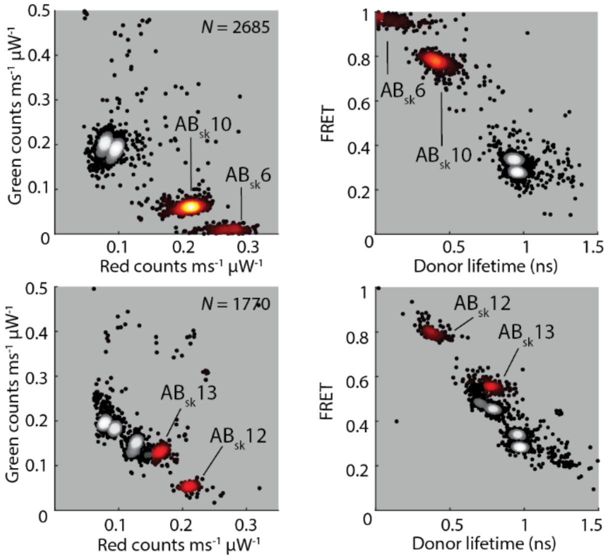

Supplementary Figure 6: Additional constructs from the AB$_{sk}$N series. Red-green and lifetime-FRET projections of data from trapped FRETfluors with design variations show clusters in different regions of the parameter space.

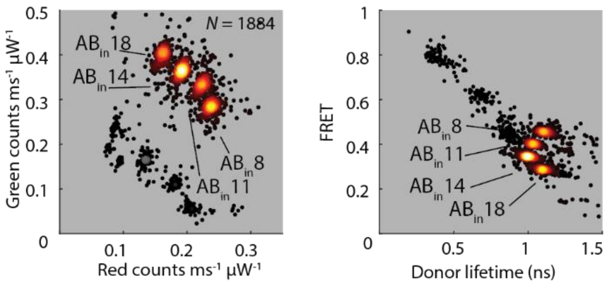

Supplementary Figure 7: Additional constructs from the $AB_{in}N$ series. Red-green and lifetime-FRET projections of data from trapped FRETfluors with design variations show clusters in different regions of the parameter space.

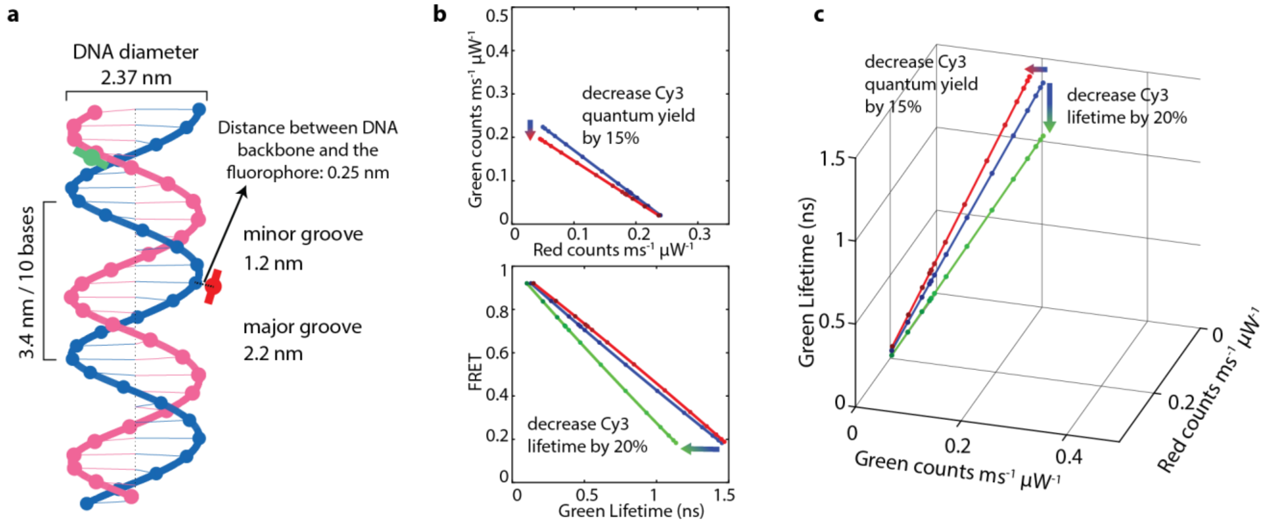

Supplementary Figure 8: Simple geometrical model for FRET on DNA. a) Cartoon of a rigid DNA double helix with doubly-tethered Cy3 and Cy5 incorporated into the backbone (shown: spacing similar to AB7). b) The calculated spectroscopic signal when the Cy3 quantum yield is reduced by 15% (red) as compared to the original quantum yield (blue). The green line represents a Cy3 lifetime reduced by 20%. c) A three-dimensional view of the model results shows that decoupled changes to quantum yield and lifetime move the FRET curve in nearly orthogonal directions in the detection parameter space.

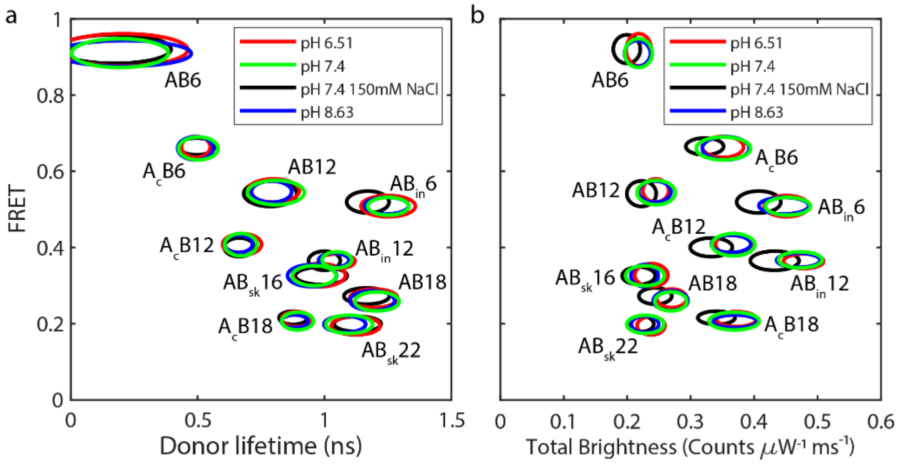

Supplementary Figure 9: FRETfluors in different salt and pH conditions. a) FRET-donor lifetime projection and b) FRET-brightness projection of cluster locations for 10 representative FRETfluors of different construct types. Cluster properties are unchanged by pH across the range 6.5-8.6, but $AB_{in}N$-type clusters show slightly reduced donor lifetime in 150 mM salt, and $AB_{in}N$-type, AB*N*-type, and A_cBN-type clusters all show slightly reduced total brightness in increased salt. The $AB_{sk}N$-type constructs appear unchanged across all conditions.

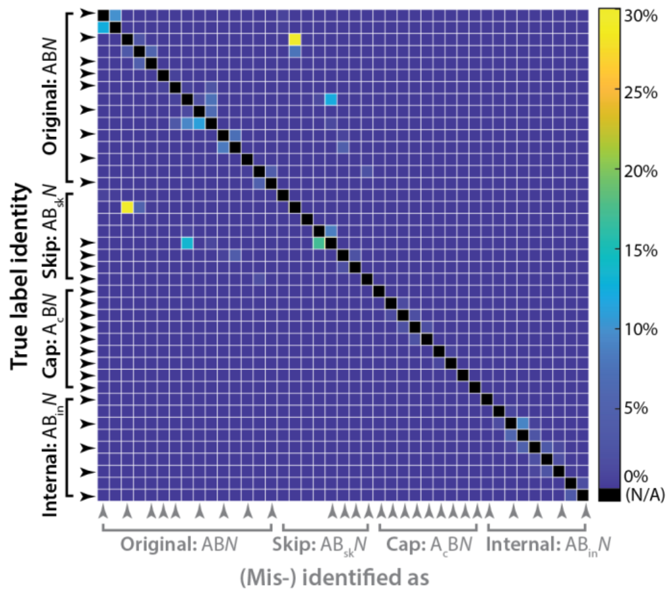

Supplementary Figure 10: Detail of the pairwise distribution analysis. Statistical selection of a near-orthogonal FRETfluor set. One-tailed Gaussian overlap between each pair of clusters was calculated for all 41 constructs. The color bar cutoff is 30%.

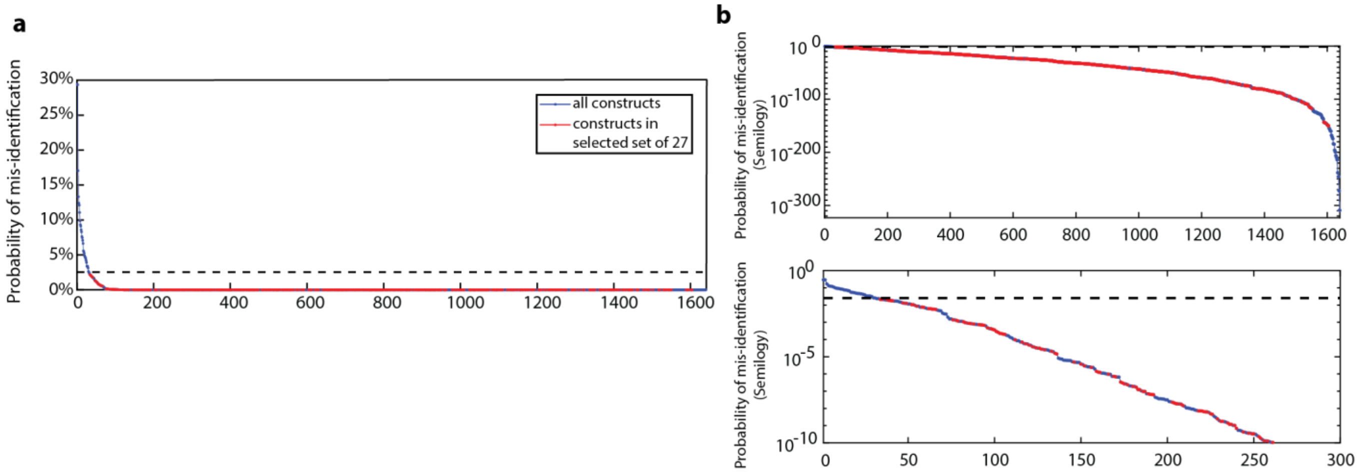

Supplementary Figure 11: Pairwise misidentification distributions. a) The ranked probabilities of pairwise misidentification for all possible FRETfluor pairs. b) Same data, y-axis shown on a log scale. Top panel is the full plot, bottom is zoomed in to show detail at higher probabilities.

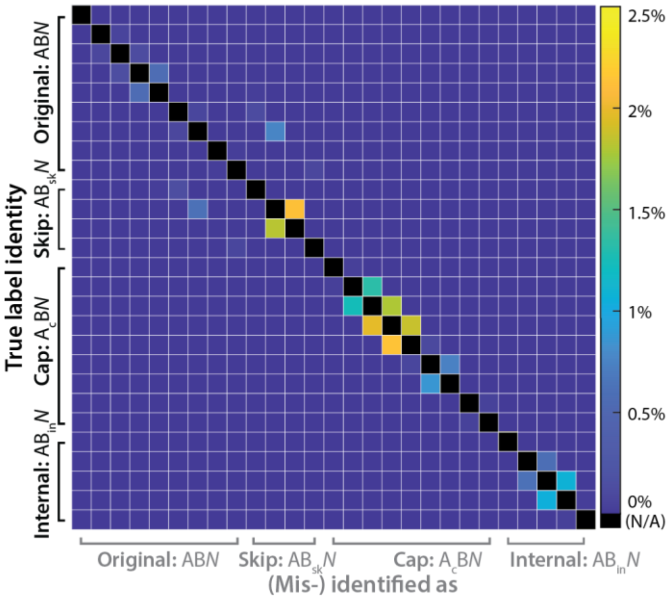

Supplementary Figure 12: Statistical selection of a near-orthogonal FRETfluor set. One-tailed Gaussian overlap between each pair of clusters was calculated for the 27 selected constructs. The color bar cutoff is 2.5%.

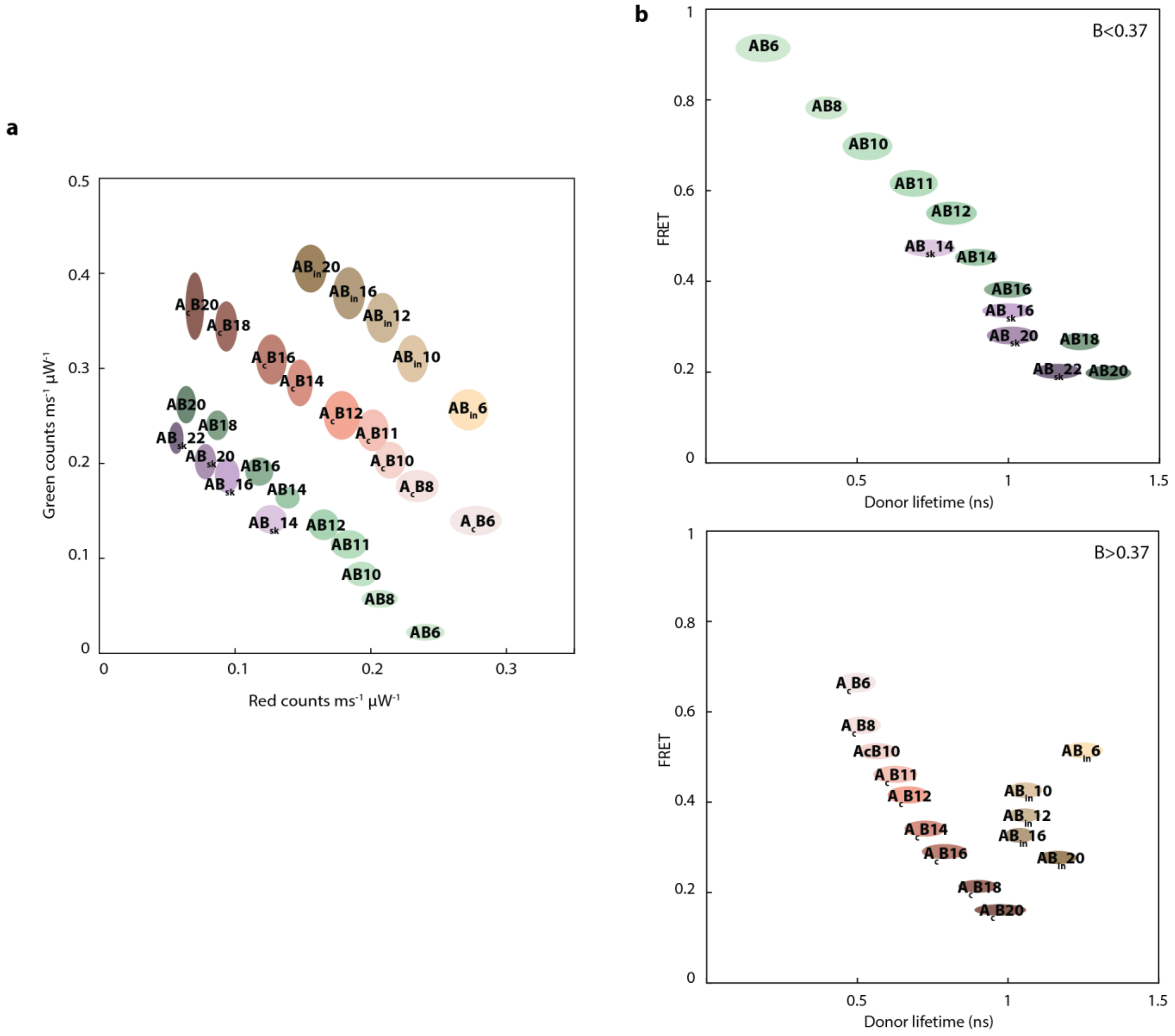

Supplementary Figure 13: Cluster locations for 27-tag FRETfluor set. a) Red-green projection of data, plotted with the calculated center and 95% confidence interval oval. b) Two brightness slices of lifetime-FRET projection of data, plotted with the calculated center and 95% confidence interval oval.

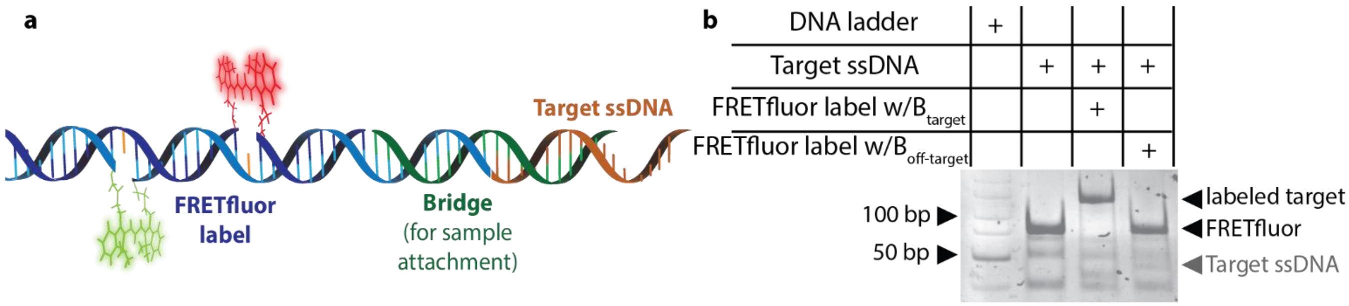

Supplementary Figure 14: Binding to ssDNA. a) Illustration of a FRETfluor construct hybridized to a ssDNA target sequence (blue: FRETfluor tag, green: labeling bridge, orange: target DNA). b) EMSA showing a mobility shift for on-target binding to a 55bp ssDNA (lane 3) as compared to free label (lane 2), but no shift for an off-target bridge sequence (lane 4). Raw / uncropped gel image is shown in Supplementary Fig. 20.

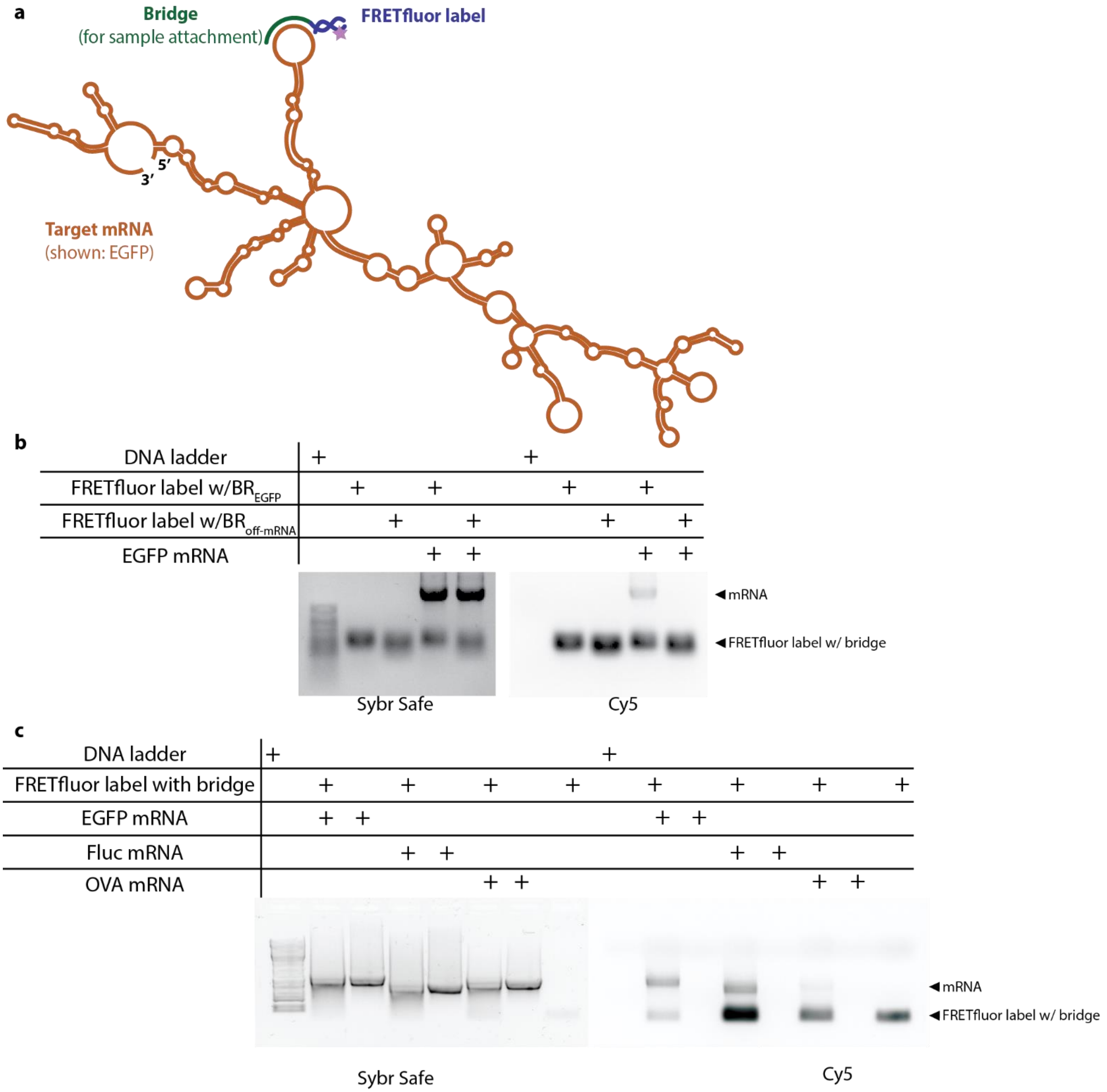

Supplementary Figure 15: FRETfluor binding to mRNA. a) Illustration of a FRETfluor label (blue) attached to a predicted hairpin loop of the EGFP mRNA through a labeling bridge (green). b) Electrophoretic mobility shift assay (EMSA) on an 1% agarose gel showing a mobility shift for on-target binding to an EGFP mRNA as compared to free label (lanes 2 and 3), but no shift for a mismatched labeling bridge. The gel was scanned in two different excitation channels: Trans UV for SyberSafe (left) and Cy5 excitation channel (right). Raw / uncropped gel image is shown in Supplementary Fig. 21. c) Electrophoretic mobility shift assay (EMSA) on an 1% agarose gel showing a mobility shift for on target binding to EGFP mRNA, FLuc mRNA and OVA mRNA as compared to the free tag itself (lanes 2, 4, 6 and lane 8) but no shift for the target itself (lanes 3, 5, 7). The gel was scanned in two different excitation channels: Trans UV for SYBR Safe (left) and Cy5 excitation channel (right). Raw / uncropped gel image shown in Supplementary Fig. 22.

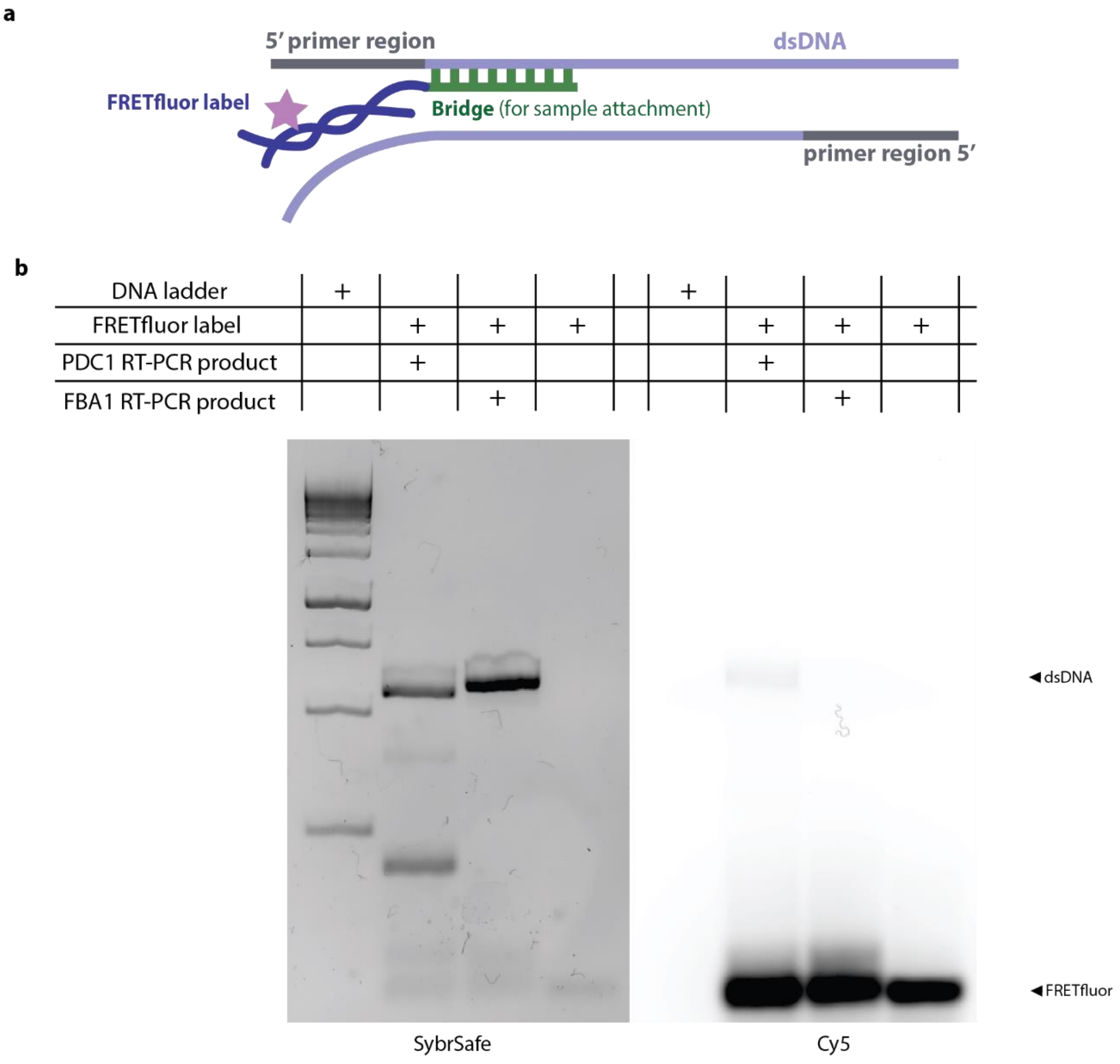

Supplementary Figure 16: FRETfluor binding to dsDNA. a) Illustration of a FRETfluor label (blue) attached to a 25 base-long targeted region of dsDNA, with a 3-base gap from the primer region on the dsDNA product (purple) through a labeling bridge (green). b) In this assay, the bridge was designed to be complementary to the PDC1 RT-PCR dsDNA. Electrophoretic mobility shift assay (EMSA) on a 3% agarose gel shows a shift upon target binding to the correct dsDNA (lane 2). No binding or shift occurs for the wrong target (lane 3), confirming labeling specificity. The gel was scanned in two different excitation channels: Trans UV for SYBR Safe (left) and Cy5 excitation channel (right). Raw / uncropped gel image shown in Supplementary Fig. 23.

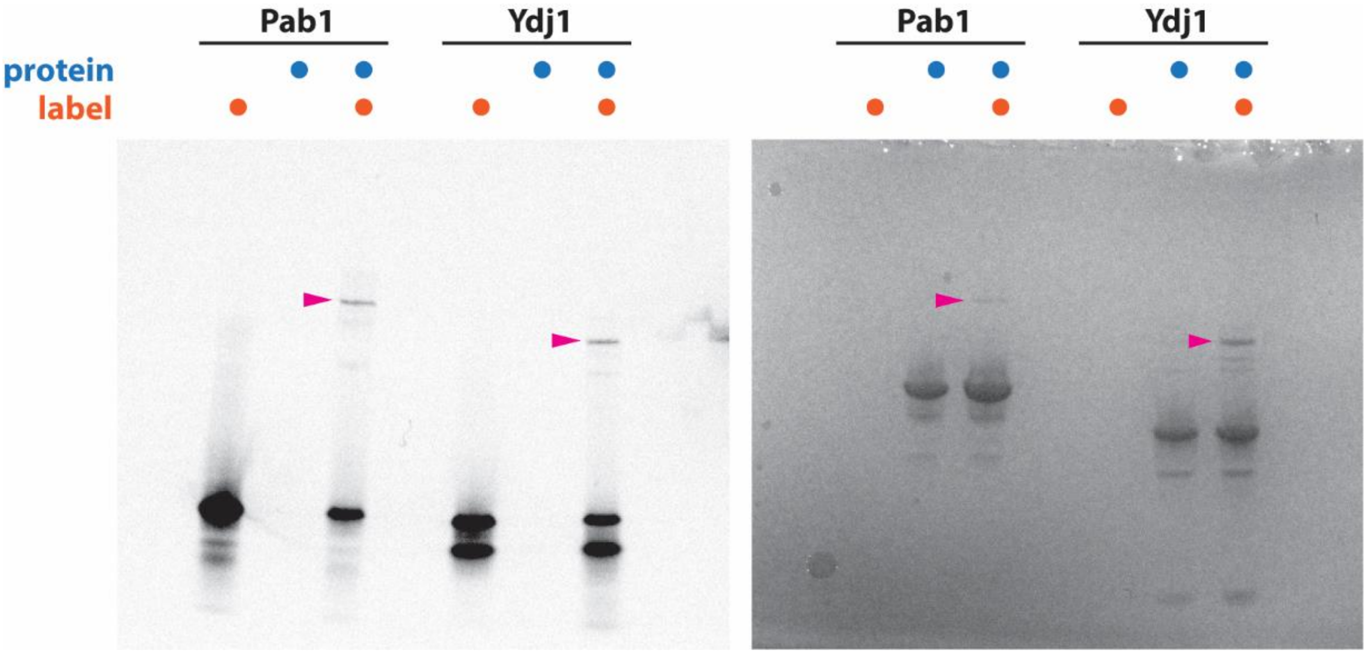

Supplementary Figure 17: FRETfluor binding to proteins. Two different single-cysteine mutant proteins (Pab1 C70A/C119A/C368A/A577C and Ydj1 C29A/C370F) reacted with FRETfluors via a covalent NHS ester-maleimide linker, as well as protein and FRETfluor alone, were compared by polyacrylamide gel electrophoresis and imaged in Cy5 fluorescence channel (left) or with white light after protein staining (right). In each protein-FRETfluor reaction, an upshifted band appears in the Cy5 and protein images (magenta arrows), indicating formation of the correct protein-FRETfluor complex. Raw / uncropped gel image shown in Supplementary Fig. 24.

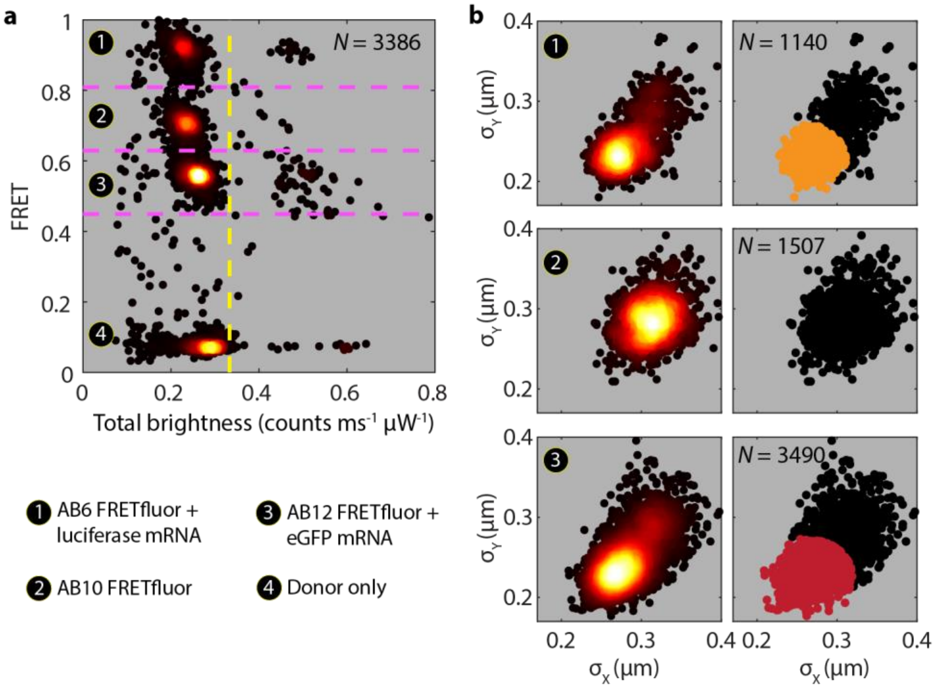

Supplementary Figure 18: FRETfluor labeling and readout of an mRNA mixture. a) Total brightness of each observed level with minimum duration 100 ms; we excluded molecules that were too bright (above the yellow line; likely dimers) from our analysis. b) For each FRETfluor, scatter plots of the standard deviations of x and y positions during trapping are shown, calculated for 1000-photon bins. For the tags bound to a target, there are two populations corresponding to bound and unbound FRETfluors, which are not observed in the AB10 control. Hard clustering for the bound populations results in the points marked in orange and red, respectively (right); these correspond to the points shown in the main text Fig. 6b.

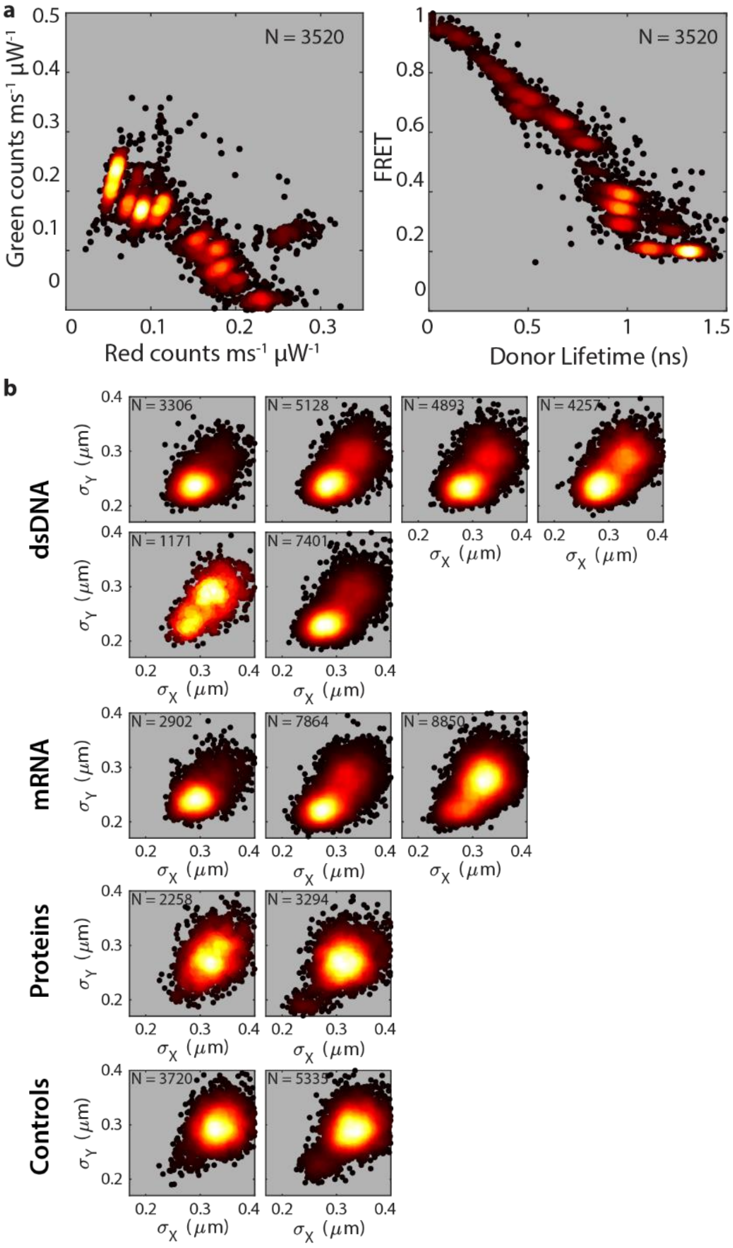

Supplementary Figure 19: FRETfluor labeling and readout of mRNA, dsDNA, and protein mixture**.** a) Red-green and lifetime-FRET projections of level-by-level data from FRETfluor-labeled dsDNA, mRNA, and proteins in a low-abundance mixture shows distinct clusters at the expected locations for each species. b) Scatter plot of standard deviation of position in *x* and *y* (calculated for 1000-photon bins) for trapped molecules of each species shows two populations in cases where the molecular weight change is significant (mRNA, dsDNA) but not for the controls. For the proteins, only one population is apparent, but it is trapped more tightly than the controls in each case. In all panels, points are colored according to the local relative scatter plot density.

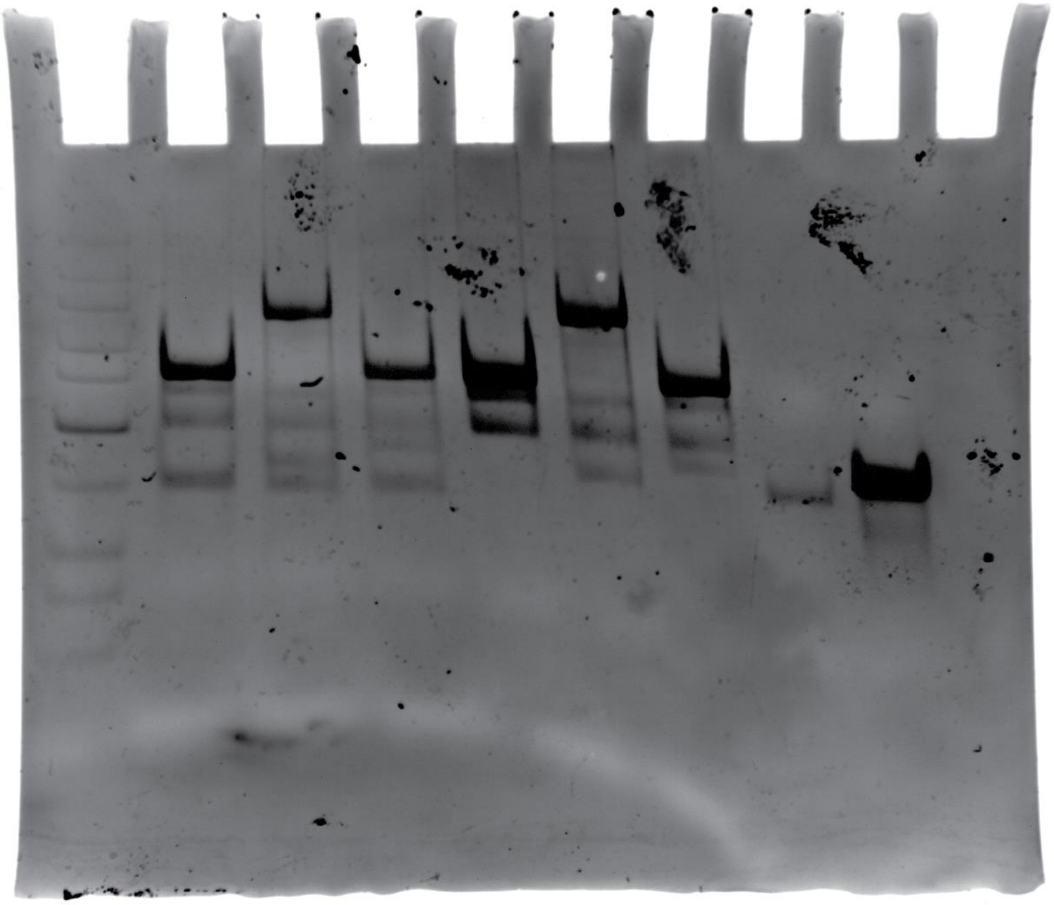

Supplementary Figure 20: Full gel scan from Supplementary Fig 14b. Relevant lanes are lane 1 to lane 4.

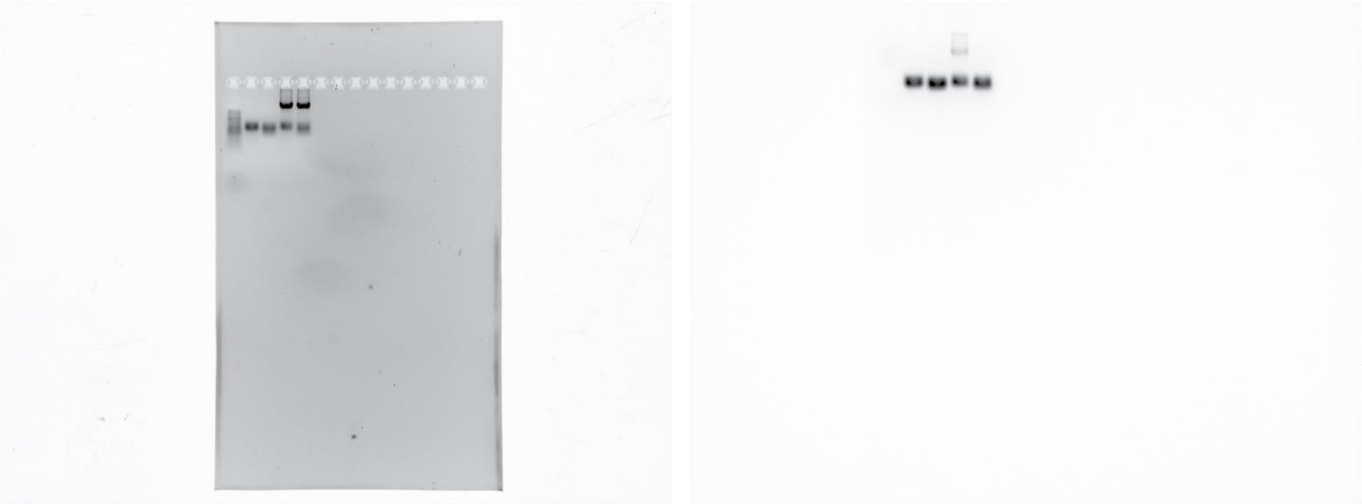

Supplementary Figure 21: Full gel scan of Supplementary Fig. 15b. Relevant lanes are lane 1 to lane 5. Left gel: SybrSafe channel, right gel: Cy5 channel.

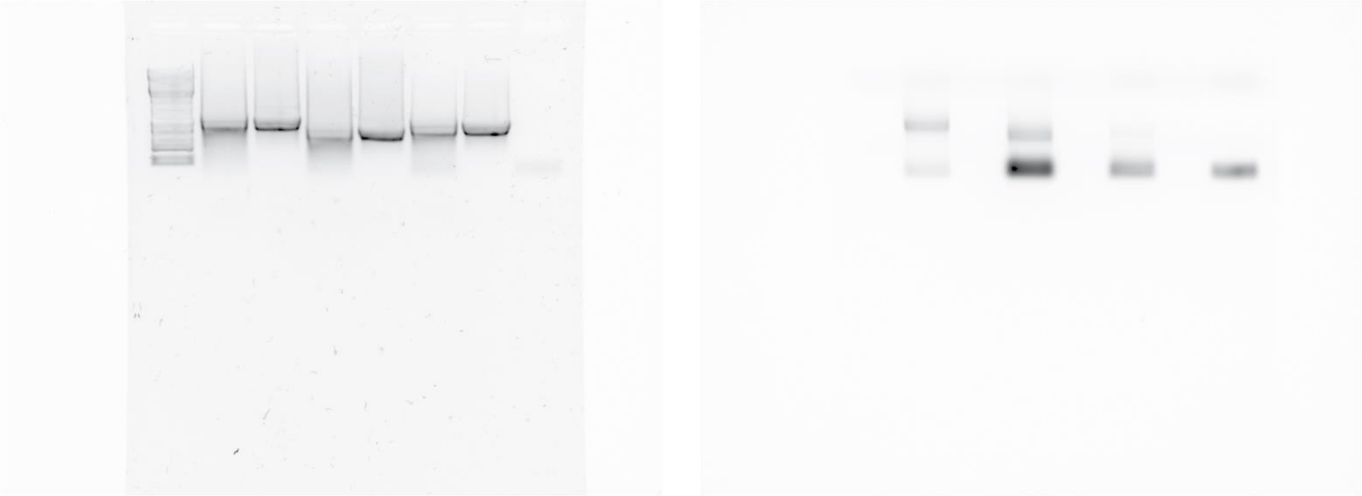

Supplementary Figure 22: Full gel scan of Supplementary Fig 15c. Relevant lanes are lane 1 to lane 8. Left gel: SybrSafe channel, right gel: Cy5 channel.

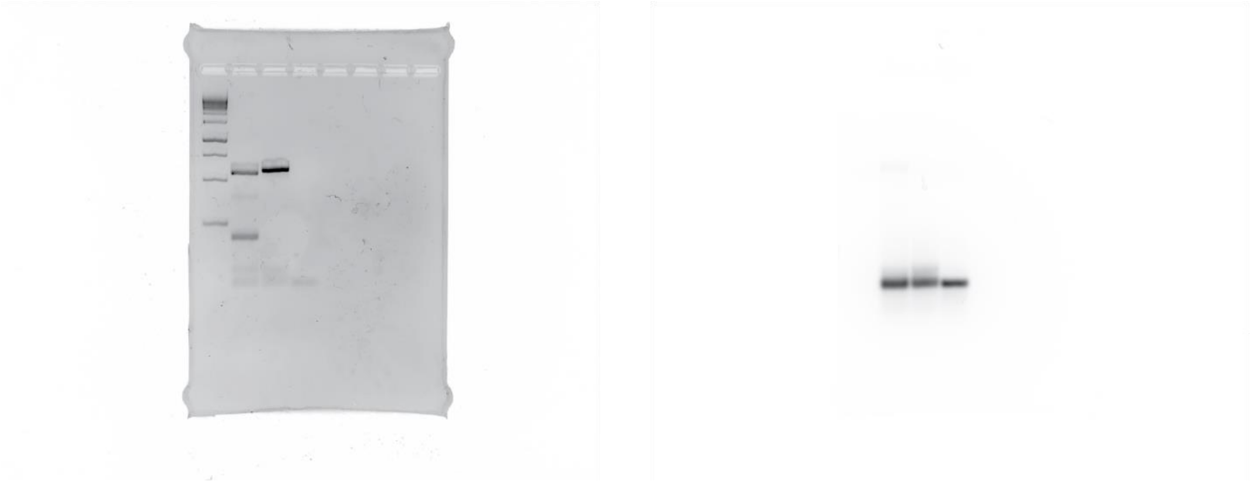

Supplementary Figure 23: Full gel scan of Supplementary Fig 16b. Relevant lanes are from lane 1 to lane 4. Left gel: SybrSafe channel, right gel: Cy5 channel.

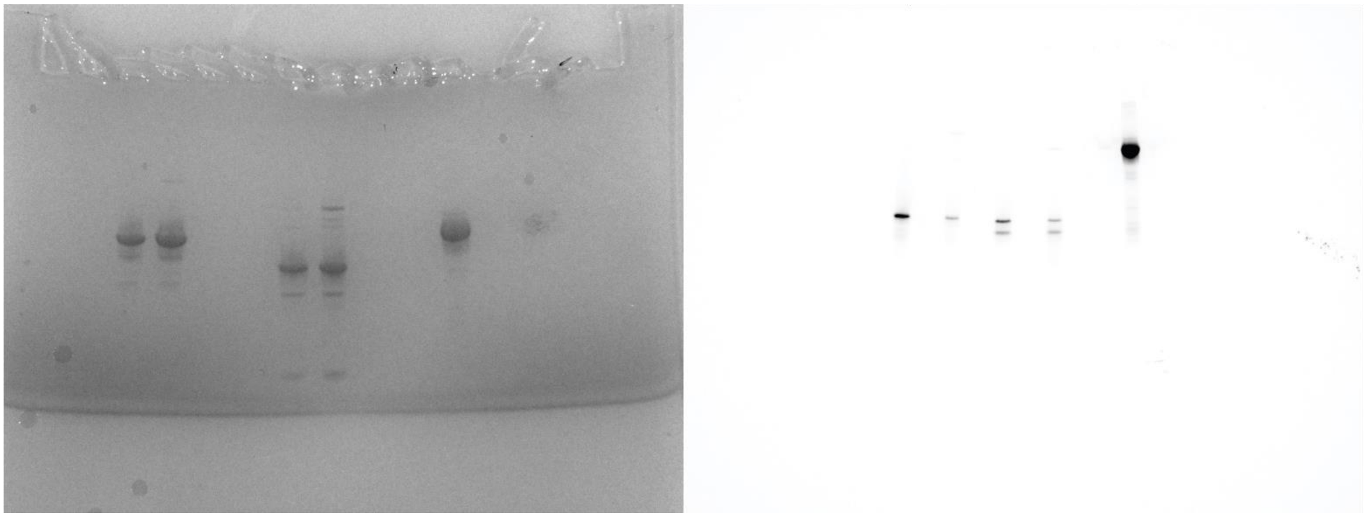

Supplementary Figure 24: Full gel scan of Supplementary Fig 17. Relevant lanes are from lane 2 to lane 8. Left gel: white light after protein staining, right gel: Cy5 channel.

Supplementary Tables

Supplementary Table 1: DNA oligomer sequences for FRETfluors and targeting bridges

A_{short}	5'	GAT GAT GTC ATC GAC /iCy3/GCG CGA TAT TCC TAC TTA TGG CGG CTC TTC CCA G	3'
A	5'	GAT GAT GTC ATC GAC /iCy3/GCG CGA TAT TCC TAC TTA TGG CGG CTC TTC CCA GCG CTA ATC ACG TTC A	3'
A_c	5'	/5Cy3/GAT GAT GTC ATC GAC /iCy3/GCG CGA TAT TCC TAC TTA TGG CGG CTC TTC CCA GCG CTA ATC ACG TTC A	3'
B6	5'	CTG GGA AGA GCC GCC ATA AGT AGG AAT /iCy5/TCG CGC CGT CGA TGA CAT CAT C	3'
B7	5'	CTG GGA AGA GCC GCC ATA AGT AGG AA/iCy5/A TCG CGC CGT CGA TGA CAT CAT C	3'
B8	5'	CTG GGA AGA GCC GCC ATA AGT AGG A/iCy5/TA TCG CGC CGT CGA TGA CAT CAT C	3'
B9	5'	CTG GGA AGA GCC GCC ATA AGT AGG /iCy5/ATA TCG CGC CGT CGA TGA CAT CAT C	3'
B10	5'	CTG GGA AGA GCC GCC ATA AGT AG/iCy5/ AAT ATC GCG CCG TCG ATG ACA TCA TC	3'
B11	5'	CTG GGA AGA GCC GCC ATA AGT A/iCy5/G AAT ATC GCG CCG TCG ATG ACA TCA TC	3'
B12	5'	CTG GGA AGA GCC GCC ATA AGT /iCy5/GG AAT ATC GCG CCG TCG ATG ACA TCA TC	3'
B13	5'	CTG GGA AGA GCC GCC ATA AG/iCy5/ AGG AAT ATC GCG CCG TCG ATG ACA TCA TC	3'
B14	5'	CTG GGA AGA GCC GCC ATA A/iCy5/T AGG AAT ATC GCG CCG TCG ATG ACA TCA TC	3'
B15	5'	CTG GGA AGA GCC GCC ATA /iCy5/GT AGG AAT ATC GCG CCG TCG ATG ACA TCA TC	3'
B16	5'	CTG GGA AGA GCC GCC AT/iCy5/ AGT AGG AAT ATC GCG CCG TCG ATG ACA TCA TC	3'
B17	5'	CTG GGA AGA GCC GCC A/iCy5/A AGT AGG AAT ATC GCG CCG TCG ATG ACA TCA TC	3'
B18	5'	CTG GGA AGA GCC GCC /iCy5/TA AGT AGG AAT ATC GCG CCG TCG ATG ACA TCA TC	3'
B19	5'	CTG GGA AGA GCC GC/iCy5/ ATA AGT AGG AAT ATC GCG CCG TCG ATG ACA TCA TC	3'
B20	5'	CTG GGA AGA GCC G/iCy5/C ATA AGT AGG AAT ATC GCG CCG TCG ATG ACA TCA TC	3'
$B_{sk}6$	5'	CTG GGA AGA GCC GCC ATA AGT AGG AAT A/iCy5/TC GCG CGT CGA TGA CAT CAT C	3'
$B_{sk}10$	5'	CTG GGA AGA GCC GCC ATA AGT AGG /iCy5/AAT ATC GCG CGT CGA TGA CAT CAT C	3'
$B_{sk}12$	5'	CTG GGA AGA GCC GCC ATA AGT A/iCy5/GG AAT ATC GCG CGT CGA TGA CAT CAT C	3'
$B_{sk}13$	5'	CTG GGA AGA GCC GCC ATA AGT /iCy5/AGG AAT ATC GCG CGT CGA TGA CAT CAT C	3'
$B_{sk}14$	5'	CTG GGA AGA GCC GCC ATA AG/iCy5/T AGG AAT ATC GCG CGT CGA TGA CAT CAT C	3'
$B_{sk}16$	5'	CTG GGA AGA GCC GCC ATA/iCy5/ AGT AGG AAT ATC GCG CGT CGA TGA CAT CAT C	3'
$B_{sk}20$	5'	CTG GGA AGA GCC GC/iCy5/C ATA AGT AGG AAT ATC GCG CGT CGA TGA CAT CAT C	3'
$B_{sk}22$	5'	CTG GGA AGA GCC /iCy5/GCC ATA AGT AGG AAT ATC GCG CGT CGA TGA CAT CAT C	3'
B_{in}	5'	TGA ACG TGA TTA GCG /3Cy3/	3'
BR_{target}	5'	AAC TGC CTG GTG ATA TGA ACG TGA TTA GCG	3'
$BR_{off\text{-}target}$	5'	ATT CCT AAG TCT GAA TGA ACG TGA TTA GCG	3'
Target(ssDNA)	5'	TAT CAC CAG GCA GTT GAC AGT GTA GCA AGC TGT AAT AGA TGC GAG GGT CCA ATA C	3'
BR_{EGFP}	5'	TGT TCT GCT GGT AGT GGT GAA CGT GAT TAG CG	3'
BR_{FLuc}	5’	CTC GGC GTA GGT GAT GTC TGA ACG TGA TTA GCG	3’

BR_{OVA}	5'	TTG TTG ATC TGG GTT GAA CGT GAT TAG CG	3'
$BR_{off\text{-}mRNA}$	5'	AAC ACA TAA ATA AAA TGA ACG TGA TTA GCG	3'
BR_{amine}	5'	/5AmMC6/TGA ACG TGA TTA GCG	3'
BR_{FBA1}	5'	CAC CCT TGA TGG AAG CAT TTT GAC CTA AAT GAA CGT GAT TAG CG	3'
BR_{CDC19}	5'	CAA CAT CGT TGG TGG TGG TAC CAG TAA ATG AAC GTG ATT AGC G	3'
BR_{ENO2}	5'	AGC AGC AGC GGC TCT AGC AGC GGC CAA ATG AAC GTG ATT AGC G	3'
BR_{TSA1}	5'	AAA GGC TAG GAC AAC GTA CTT ACC CAA ATG AAC GTG ATT AGC G	3'
BR_{RPL5}	5'	CTT CTC TTC TTC TTC TGA AAG GAG TAA ATG AAC GTG ATT AGC G	3'
BR_{SSA3}	5'	CCA ACC TAT TAT CAA AAT CTT CAC CAA ATG AAC GTG ATT AGC G	3'

KEY for Supplementary Table S1:

X:	Base is opposite to Cy3 after annealing
XX:	No base immediately opposite to Cy3, so the Cy3 will be more exposed to solvent
X/iCy5/X:	No base immediately opposite to Cy5, so the Cy5 will be more exposed to solvent
XXX/XXX:	Complementary sequences are shown in the same color
XXX:	Complementary to the target mRNA, also highlighted in gray (see SI Note 8)
XXX:	Complementary to the target dsDNA

Supplementary Table 2: Lifetime fitting of donor-only constructs

Construct (population)	Brightness (counts μs^{-1} μW^{-1})	1-exp Lifetime (ns)	2-exp Lifetimes #1 (% population; ns)		2-exp Lifetimes #2 (% population; ns)	
**AB0**	0.31 ± 0.012	1.60 ± 0.03	41% ± 4.0%	0.69 ± 0.05	59% ± 4.0%	1.80 ± 0.03
**AB$_{sk}$0**	0.26 ± 0.007	1.25 ± 0.03	37% ± 4.5%	0.64 ± 0.07	63% ± 4.5%	1.41 ± 0.05
**AB$_{in}$0** (AB$_{in}$0)	0.56 ± 0.015	1.51 ± 0.03	44% ± 1.1%	0.65 ± 0.02	56% ± 1.1%	1.76 ± 0.02
(bridge Cy3 only)	0.24 ± 0.010	1.40 ± 0.03	47% ± 4.2%	0.55 ± 0.03	53% ± 4.2%	1.65 ± 0.04
**A$_c$B0** (A$_c$B0)	0.40 ± 0.011	1.07 ± 0.03	59% ± 1.4%	0.50 ± 0.03	41% ± 1.4%	1.36 ± 0.02
(cap Cy3 only)	0.17 ± 0.007	0.52 ± 0.02	70% ± 11%	0.32 ± 0.06	30% ± 11%	0.72 ± 0.08
(Q-A$_c$B0)	0.32 ± 0.013	0.82 ± 0.03	62% ± 5%	0.45 ± 0.04	38% ± 5%	1.07 ± 0.04

Supplementary Table 3: FRETfluor cluster data

**Tag**	**FRET** unitless, [0,1]	**Donor Lifetime** (ns)	**Red Brightness** (counts μs^{-1} μW^{-1})	**Green Brightness** (counts μs^{-1} μW^{-1})
**AB6**	0.916 ± 0.016	0.187 ± 0.045	0.240 ± 0.007	0.022 ± 0.004
AB7	0.904 ± 0.016	0.270 ± 0.049	0.252 ± 0.010	0.027 ± 0.005
**AB8**	0.783 ± 0.013	0.398 ± 0.035	0.207 ± 0.007	0.057 ± 0.005
AB9	0.743 ± 0.012	0.486 ± 0.050	0.210 ± 0.008	0.073 ± 0.006
**AB10**	0.698 ± 0.015	0.534 ± 0.041	0.193 ± 0.006	0.084 ± 0.006
**AB11**	0.616 ± 0.015	0.687 ± 0.040	0.184 ± 0.007	0.115 ± 0.007
**AB12**	0.550 ± 0.013	0.813 ± 0.042	0.165 ± 0.006	0.135 ± 0.008
AB13	0.488 ± 0.011	0.797 ± 0.035	0.136 ± 0.006	0.143 ± 0.009
**AB14**	0.453 ± 0.009	0.895 ± 0.035	0.138 ± 0.004	0.167 ± 0.006
AB15	0.482 ± 0.017	0.839 ± 0.042	0.148 ± 0.007	0.159 ± 0.010
**AB16**	0.381 ± 0.009	1.001 ± 0.040	0.118 ± 0.005	0.191 ± 0.007
AB17	0.342 ± 0.012	1.034 ± 0.038	0.107 ± 0.006	0.207 ± 0.009
**AB18**	0.266 ± 0.009	1.238 ± 0.033	0.087 ± 0.004	0.240 ± 0.008
AB19	0.214 ± 0.007	1.234 ± 0.030	0.067 ± 0.004	0.244 ± 0.013
**AB20**	0.197 ± 0.008	1.333 ± 0.038	0.064 ± 0.004	0.262 ± 0.010
AB$_{sk}$6	0.962 ± 0.012	0.125 ± 0.047	0.272 ± 0.010	0.011 ± 0.003
AB$_{sk}$10	0.776 ± 0.014	0.417 ± 0.033	0.210 ± 0.008	0.061 ± 0.004
AB$_{sk}$12	0.551 ± 0.008	0.676 ± 0.023	0.149 ± 0.003	0.121 ± 0.004
AB$_{sk}$13	0.480 ± 0.009	0.699 ± 0.025	0.123 ± 0.004	0.133 ± 0.004
**AB$_{sk}$14**	0.472 ± 0.009	0.741 ± 0.042	0.126 ± 0.006	0.141 ± 0.007
**AB$_{sk}$16**	0.334 ± 0.008	0.998 ± 0.042	0.094 ± 0.005	0.188 ± 0.009
**AB$_{sk}$20**	0.279 ± 0.010	1.011 ± 0.041	0.078 ± 0.004	0.202 ± 0.009
**AB$_{sk}$22**	0.200 ± 0.008	1.164 ± 0.038	0.057 ± 0.003	0.227 ± 0.008
**A$_c$B6**	0.665 ± 0.011	0.498 ± 0.031	0.277 ± 0.009	0.140 ± 0.008
**A$_c$B8**	0.571 ± 0.010	0.515 ± 0.031	0.234 ± 0.008	0.176 ± 0.008
**A$_c$B10**	0.513 ± 0.009	0.564 ± 0.033	0.214 ± 0.006	0.203 ± 0.010
**A$_c$B11**	0.462 ± 0.009	0.626 ± 0.036	0.202 ± 0.006	0.235 ± 0.011
**A$_c$B12**	0.416 ± 0.009	0.669 ± 0.034	0.179 ± 0.007	0.251 ± 0.013
**A$_c$B14**	0.342 ± 0.009	0.725 ± 0.034	0.148 ± 0.005	0.285 ± 0.012
**A$_c$B16**	0.291 ± 0.008	0.789 ± 0.037	0.127 ± 0.006	0.309 ± 0.013
**A$_c$B18**	0.214 ± 0.007	0.900 ± 0.032	0.094 ± 0.004	0.344 ± 0.013
**A$_c$B20**	0.161 ± 0.006	0.975 ± 0.043	0.070 ± 0.003	0.365 ± 0.018
**AB$_{in}$6**	0.515 ± 0.009	1.249 ± 0.033	0.272 ± 0.007	0.256 ± 0.011
AB$_{in}$8	0.457 ± 0.006	1.132 ± 0.019	0.239 ± 0.005	0.284 ± 0.007
**AB$_{in}$10**	0.427 ± 0.009	1.057 ± 0.028	0.231 ± 0.006	0.310 ± 0.012
AB$_{in}$11	0.403 ± 0.005	1.047 ± 0.018	0.220 ± 0.004	0.327 ± 0.007
**AB$_{in}$12**	0.372 ± 0.007	1.054 ± 0.027	0.209 ± 0.006	0.353 ± 0.013
AB$_{in}$14	0.344 ± 0.005	1.017 ± 0.019	0.203 ± 0.004	0.387 ± 0.008
**AB$_{in}$16**	0.327 ± 0.008	1.038 ± 0.024	0.184 ± 0.006	0.379 ± 0.014

$AB_{in}18$	0.290 ± 0.005	1.088 ± 0.017	0.159 ± 0.004	0.388 ± 0.008
**AB_{in}20**	0.278 ± 0.007	1.166 ± 0.031	0.156 ± 0.006	0.405 ± 0.013

*FRETfluor labels in **BOLD** are part of 27-component mixture

Supplementary Table 4: Heterogeneous mixture components and labels

Target	Target type	FRETfluor label	Bridge name	Notes
FBA1	dsDNA	AB6	BR_{FBA1}	RT-PCR product for fructose 1,6-bisphosphate aldolase
EGFP	mRNA	AB8	BR_{EGFP}	mRNA for enhanced Green Fluorescent Protein
CDC19	dsDNA	AB10	BR_{CDC19}	RT-PCR product for pyruvate kinase
ENO2	dsDNA	AB11	BR_{ENO2}	RT-PCR product for phosphopyruvate hydratase
TSA1	dsDNA	AB12	BR_{TSA1}	RT-PCR product for thioredoxin peroxidase
RPL5	dsDNA	AB14	BR_{RPL5}	RT-PCR product for a ribosomal 60S subunit
FLuc	mRNA	AB16	BR_{FLuc}	mRNA for firefly luciferase protein
Ydj1	protein	AB18	BR_{amine}	Cytosolic class A J-domain protein
OVA	mRNA	AB20	BR_{OVA}	mRNA for ovalbumin
Pab1	protein	A_cB6	BR_{amine}	Poly(A) binding protein from yeast
SSA3	dsDNA	$AB_{sk}16$	BR_{SSA3}	RT-PCR product for heat shock protein Hsp70
control	-	$AB_{sk}20$	None	FRETfluor only
control	-	$AB_{sk}22$	None	FRETfluor only

Supplementary Table 5: Target genes and RT-PCR primers

Gene	Forward Primer (5'-3')	Reverse Primer (5'-3')	Reference Sequence (S288C, coding DNA)[1,2]
FBA1	TCGCTGGTAAGGGTATCTCTAA	CCGTGGAAGACCAAGAACAA	https://www.yeastgenome.org/locus/S000001543/sequence
CDC19	ACACCAAGGGTCCAGAAATC	TCACCTCTGGCAACCATAAC	https://www.yeastgenome.org/locus/S000000036/sequence
ENO2	CTAACGCTATCTTGGGTGTCTC	GGAGTGGTACATGTCAGCTAAT	https://www.yeastgenome.org/locus/S000001217/sequence
TSA1	GACGAAGTCTCCTTGGACAAATA	GGCAGCTTCGAAGTATTCCT	https://www.yeastgenome.org/locus/S000004490/sequence
RPL5	CTCTGCTTACTCCTCTCGTTTC	CTGGGTCAATTTCTTCGGTTTC	https://www.yeastgenome.org/locus/S000006052/sequence
SSA3	CCGCAGGAGACACTCATTTA	CCTTCGAGAGCCTACCTTTATC	https://www.yeastgenome.org/locus/S000000171/sequence

Supplementary Notes

Supplementary Note 1: Sequence- and attachment-dependent photophysics of Cy3

Physical and chemical properties of the nano-environment of a fluorophore, and in particular the resulting dielectric environment, strongly influence fluorophore photophysics. Different solvents, substrates, and attachment chemistries therefore can alter observed photophysical properties from their baseline values; conversely, small modifications to the local environment can be used to intentionally alter these properties, as we have done in this work. Traditionally, the brightness or quantum yield Φ of a fluorophore is expected to change proportionally with the observed lifetime τ, since

$$\tau = \frac{1}{k_r + k_{nr}} \quad (1)$$

and

$$\Phi = \frac{k_r}{k_r + k_{nr}} \quad (2)$$

and the native radiative rate k_r of a fluorophore is assumed to be constant while only k_{nr} changes. However, if the dielectric environment of the fluorophore chances due to changing exposure to solvent or to changes in the chemical surroundings, k_r can also change, decoupling τ and Φ.[1]

We measured the brightness and fluorescence lifetime of Cy3 alone for each type of construct used in this work. In all naming conventions used here, a "0" denotes the lack of a Cy5 dye on the B strand. Supplementary Figure 1 shows aggregated single-molecule data for Cy3-only complexes. Constructs with two Cy3s show a minimum of three populations (one each for the Cy3s, one for the combined signal). We observe that the skipped constructs $AB_{sk}0$ show slightly reduced lifetime and brightness, possibly due to additional conformational flexibility and solvent exposure. The additional Cy3 on the bridge is also dimmer with a shorter lifetime, but clearly distinct from the $AB_{sk}0$ construct. The total $AB_{in}0$ signal is a near-perfect sum of the bridge Cy3 and the original AB0.

The A_cB0 construct is more complicated, likely due to base stacking or other conformational changes induced by the Cy3 at the 5′ end of the A strand. A very dim population appears to correspond to the cap Cy3 only, as evidenced by transitions to and from the total A_cB0 signal population. The additional population is labeled with a "Q" because it appears to be a slightly quenched version of the A_cB0 population, although the reason for this population is not understood. We do not see FRETfluor signals for the $A_cB\mathit{N}$ series that are consistent with the Q population acting as a donor.

All donor-only population means and standard deviations for brightness and 1-exp lifetime fits, along with the 2-exp lifetime fits for comparison, are provided in Supplementary Table 2. For the 2-exp lifetimes, photons from all levels in the 1-exp populations shown in the scatter plots were aggregated into a single decay for each data set, for which a 2-exp lifetime fit was performed. Lifetimes, standard deviations, and fractional populations are given for a set of fits across a minimum of seven data sets each (max 15); outliers would have been excluded but the 2-exp results were consistent and did not contain outliers.

Critically, we observe that τ and Φ indeed appear to vary independently for most Cy3-only populations. This decoupling is useful in the context of this work, as explained in Supplementary Note 4 and Supplementary Fig. 8, because it allows the FRET curve to be moved in different directions.

Supplementary Note 2: Raw trapping events

Supplementary Fig. 2 shows raw trapping events from a mixture including 27 FRETfluor labels. As described in *Methods*, change-point detection is used to identify all levels in the data on the basis of maximum likelihood changes in brightness. After clustering of the levels, the main populations for each of the labels can be identified, and those cluster locations and variance (SI Table S3) are used to classify all subsequent data. Events are automatically classified based on the type(s) of levels passing a duration filter (> 150 ms) that they contain:

Most events contain a single state, but some also contain "unidentified" levels that do not match any cluster. We found that many (if not most) unidentified levels within assigned events represent "allowed transition levels" for that FRETfluor species, even though we did not explicitly analyze these levels' clusters. This is evident because the "allowed transition" states (a) sometimes show up multiple times within one event and (b) have combinations of measured parameters that are plausibly explained for that construct (for example, by blinking of a donor). Photophysical parameters within individual levels are generally stable over time. In cases where non-allowed transitions are observed without a background level indicating an empty trap between events, it is likely that one trapped molecule was randomly replaced with another during the event.

Here we highlight a few selected events to illustrate these effects:

For example, in the second row between 120-123 seconds, which shows an event from the tag AB8, the donor and acceptor are on during the event. Then, at 123 sec, the acceptor photobleaches, dropping the red brightness to nearly zero, and the green brightness level goes up. The new green-only level is consistent with the properties of the AB0 donor only construct as characterized in Supplementary Fig. 1.

Another example is the event in the first row at 15-25 seconds, which shows an event from the tag $AB_{in}10$, where both donors are on during the first part of the event. Then at 21 sec, the donor on the bridge blinks, leaving the red brightness the same and the green brightness lower than before. The spectroscopic information for the second part of this event is similar to an AB10 label. However, since there is no gap between these two states, we conclude that this blinking state also belongs to tag $AB_{in}10$.

In the second row at 62-70 seconds, the first part of the event shows both donors on A_cB10 are on, while the second part (at 65 sec) shows that the donor on the cap is off. This leaves the green brightness lower while the red brightness does not change much. The spectroscopic information for the second part of this event is also similar AB10. However, since the gap between these two states is extremely short, we also assign this level to tag A_cB10.

There are also several events indicating that there is very weak energy transfer between the second donor and the acceptor on the main strand. In the second row between 10-22 seconds, the first part of the event shows both donors and the acceptor for $AB_{in}20$ are all on while the second part (at 20 sec) shows that the donor on the bridge has blinked off. The photobleaching of the donor on the bridge leaves both the red and green brightness lower than before.

Supplementary Note 3: Trapping event rate and limit of detection

To determine the lowest working concentrations at which FRETfluors could reasonably be detected, we characterized the trapping event rate in our ABEL trap setup. We acquired data for two separate dilution series of the FRETfluor AB12. We tested concentrations from 5 pM down to 5 fM (Exp 1: 50 fM, 1 pM, 5 pM; Exp 2: 5 pM, 500 fM, 100 fM, 50 fM, 25 fM). We collected three 5-minute long data sets for each sample and rinsed the sample cell thoroughly between uses with DI water (>5x sequential volume rinses each). In the first experiment (Supplementary Fig. 3a), samples were tested in order of increasing concentration. In the second experiment (Supplementary Fig. 3b), samples were tested in order of decreasing concentration. The consistent results obtained from these two experiments suggests that washing out the ABEL trap cell effectively removes the previous sample to below detectable concentrations.

In analyzing the data, trapping events were defined as consecutive 10-ms bins above background with a minimum total level duration of 150 ms and brightness and lifetime values within 3.5 standard deviations of the AB12 population (see Supplementary Table 3). Zero events were recorded across buffer-only data sets. Linear fits with intercept = 0 were fit to each data set, with slopes ~0.1 events s^{-1} pM^{-1}.

Assuming Poisson-distributed trapping events, an expected value of 3 events during the measurement window is required to reach 95% confidence of nonzero rate (5% probability of recording 0 counts):

$$P(0) = \frac{\langle\#\text{events}\rangle^{0} e^{\langle\#\text{events}\rangle}}{\langle\#\text{events}\rangle!} = 0.05 \tag{3}$$

This constraint yields ⟨#events⟩=3 counts. Therefore, in a 15 min measurement window (t = 900 s), the minimum statistically detectable concentration would be:

$$C = \frac{\langle\#events\rangle}{kt} = \frac{3\text{ counts}}{(0.1\,{}^{\text{counts}}/_{\text{s·pM}})(900\text{ s})} = 33\text{ fM} \tag{4}$$

We routinely work at hundreds of fM; in the present work the 27-component FRETfluor mixture data was acquired at ~70 fM per component, and the 13-component mixed biomolecular sample was acquired at ~350 fM per component.

Supplementary Note 4: Simulation of energy transfer between Cy3 and Cy5 on dsDNA

To model the expected magnitude of changes to different parameters in the spectroscopic output of FRETfluors due to changes in donor or acceptor photophysical properties, we created a simple model of FRET for doubly-tethered Cy3 and Cy5 on double-stranded DNA (dsDNA) after published works.[3–5]

Supplementary Fig. 8a shows the simple geometrical model of a rigid DNA double helix with doubly-tethered Cy3 and Cy5 incorporated into the backbone. B-form DNA parameters were taken from Ref 6.[6] The diameter of the DNA is 2.37 nm, and the gap between base pairs is 0.34 nm (10 bp/turn). The distance between the DNA backbone and the fluorophore was set at 0.25 nm. The angular offset between the 3'-5' and the 5'-3' strand (-127°) is set by the minor groove height (1.2 nm) and helicity.

For simplicity, we assumed that both dyes could rotate freely ($\kappa^2 = 2/3$).[3] While some constriction of the dipole cone angles in FRETfluors is anticipated due to the backbone attachment of the Cy dyes, here our observed FRET curve for sequential constructs (for example, the AB*N* series shown in Fig. 3a) does not exhibit significant helix-dependent orientation effects. This result suggests that the dyes have relatively mobile orientation, so while κ^2 may not be exactly 2/3, it does not vary substantially across constructs and is therefore likely to be close to 2/3.

To create different constructs that were representative of different dye spacings in our FRETfluors, the Cy3 (donor) was kept at the same location for each construct and the Cy5 (acceptor) was moved to different locations along the opposite strand.

Dye photophysical parameters were assigned according to measured values when possible. For example, we measured the single-exponential fit to the lifetime decay for Cy3 doubly-tethered to the DNA backbone at the single molecule level as 1.6 ns, as shown in Supplementary Fig. 1. Other parameters: Cy5 lifetime (1.7 ns), quantum yields Φ_{Cy3}= 0.15 and Φ_{Cy5}= 0.27.[7,8]

Briefly, we simulated a coupled energy transfer model where the time evolution of the probability of exciton residence on each fluorophore following absorption, $p = [p_{Cy3}(t), p_{Cy5}(t)]$ by one or the other fluorophore, $dp(t)/dt$, may be described by the master equation:

$$\frac{d\boldsymbol{p}(t)}{dt} = \boldsymbol{M}\boldsymbol{p}(t) \tag{5}$$

M is an excitation transition matrix with off-diagonal elements representing the pairwise energy transfer rates, k_{ji}, (row *i*, column *j*) between donor *j* and acceptor *i* pigments. Entries on the diagonal indicate the total rate of energy loss at that fluorophore, including both energy transfer to the other pigments and the rate of fluorescent emission at that site, k_{ii}^{10}:

$$M_{ii} = -\left(k_{ii}^{10} + \sum_{i,j\neq i} k_{ji}\right) \tag{6}$$

To calculate initial values for the energy transfer rates, we used the Förster energy transfer equation[3,9] using absorption and emission spectra for Cy3 and Cy5:

$$k_{DA} = \frac{k_D^{10}\Phi_D\kappa^2}{R_{DA}^6}\left(\frac{9000\ln 10}{128\pi^5 N_A n^4}\right)\frac{\int Em_D\varepsilon_A\lambda^2 d\lambda}{\int Em_D\lambda^{-2}d\lambda} \tag{7}$$

For each initial condition $p(0)$ = [0, 1] and $p(0)$ = [1, 0], we can find the total probability of fluorescence emission from site *i* given excitation at site *j* by integrating over a long time (here: ~10 ns):

$$P_i^j \equiv \int_0^{+\infty} p_i^j(t) k_i^{10}\, \Phi_i dt \tag{8}$$

where Φ_i is the quantum yield of pigment *i*. The total probability of emission from fluorophore *i* given excitation at either fluorophore *j*, P_i^j, is then given according to the relative probabilities of each initial condition (determined by the absorbance probability at the excitation wavelength, 532 nm, for each fluorophore)

$$P_i = \sum_j A_j P_i^j \tag{9}$$

where A_j is the absorption probability at 532 nm for fluorophore *j*. The total probability of emission from fluorophore *i*, P_i, is directly proportional to the photon emission rate, or brightness B_i, from that fluorophore, by some unknown constant (*vide infra*). The predicted emission spectrum is a weighted sum of the individual fluorophore spectra, where the relative brightness of each pigment is used to weigh its contribution to the spectrum:

$$Em(\lambda) = \frac{\sum_i B_i Em_i(\lambda)}{\sum_i B_i} \tag{10}$$

This spectrum is separated into two spectral windows that encompass the red channel and the green channel, respectively. In order to allow our simulation results to be compared to measured data, we multiplied each brightness channel by the transmission profile of our emission filters (see *Methods*). In total, this translated to 71.43% throughput for the green channel, *Em*(green), and 46.18% throughput for the red channel, *Em*(red). Next, we scaled these unitless brightnesses to be comparable to our experimental brightness data for the AB*N* constructs using an approximate scaling factor (3 counts / 10 ms / µW). These became the simulation outputs for green and red channel brightnesses, "Green counts" and "Red counts" per Supplementary Fig. 8b (top panel).

As with our experimental data, FRET values (Supplementary Fig. 8b, bottom panel) were calculated according to an uncorrected ratio between the red channel and the sum over both channels:

$$FRET = \frac{\Sigma Em(red)}{\Sigma Em(red) + \Sigma Em(green)} \tag{11}$$

Finally, we predicted the observable single-exponential fluorescence lifetime of the donor by constructing the fluorescence decay of that state, $g_{donor}(t)$, and then fitting a single exponential decay function to find the apparent lifetime (see Supplementary Fig. 8b, bottom panel):

$$g_{donor}(t) = \sum_j k_i^{10} \Phi_i \left(\frac{\sum_j A_j p_i^j(t)}{\sum_j A_j} \right) \tag{12}$$

We calculated green and red brightness, green lifetime, and FRET values for complexes with spacings from 6 to 20 bp.

Supplementary Note 5: Effect of changing donor photophysical properties on FRET

We used our FRET simulation as described in Supplementary Note 4 above to model the expected magnitude of changes in spectroscopic signals we might measure for certain changes in photophysical properties of the donor Cy3. We separately modeled changes to the lifetime, τ, and quantum yield, Φ, of the donor, since our measurements of donor properties of different constructs indicated that changes in these variables across different constructs were not perfectly coupled (see Supplementary Note 1).

Per Supplementary Note 4, we experimentally observed donor brightnesses as low as 50% (for $A_{cap\text{-}only}B0$) compared to the brightest donor construct (AB0). A subtler reduction of ~15% brightness was observed for $AB_{sk}0$ as compared to AB0. Here, we take changes in brightness to reflect changes in quantum yield. For lifetimes, we observed reductions by up to a factor of three (for $A_{cap\text{-}only}B0$ as compared to AB0), and at minimum a 20% reduction ($AB_{sk}0$ as compared to AB0).

We modeled these minimal changes in Φ (15% reduction, red) and τ (20% reduction, green) using the simulation described in Supplementary Note 4 above to determine the predicted changes in our measured signals as compared to the original values (blue). As expected, both changes are most evident when FRET is low, and produce corresponding reductions in the observed brightness and lifetime for the lowest FRET states of approximately 15% and 20% for quantum yield and green lifetime, respectively (Supplementary Fig. 8b). We therefore expected that if populations with lower FRET could be clustered tightly within a few % or less, populations from the original constructs could be easily differentiated from the constructs with the modified donor properties.

These results also illustrate that the effects of changing Φ and τ are nearly orthogonal for the two data projections used throughout this work: The top panel is a brightness-brightness projection for the two color channels, while the bottom panel is a FRET- τ_{Green} projection. We note that the reduced Cy3 lifetime is apparent as a shifted green line only in the bottom panel of Supplementary Fig. 8b, and overlaps almost perfectly with the original blue line in the top panel (not shown due to overlap). Similarly, the reduced Cy3 quantum yield creates an obviously shifted red line top panel, but overlaps almost perfectly with the original blue line in the bottom panel.

In the three-dimensional view shown in Supplementary Fig. 8c, the near-orthogonality of these motions in Gr-R-τ space is clear. As discussed in Supplementary Note 4, traditionally Φ and τ are expected to be perfectly correlated under the assumption that the native radiative rate of a fluorophore does not change.[10] Here, our simulations illustrate the advantage of a situation where Φ and τ are not perfectly coupled: independently changing donor properties can move the highly correlated FRET curve around the multidimensional parameter space, providing opportunities to create many distinct constructs.

The shifts in the simulated FRET curve at low FRET both (1) roughly correspond to the donor-only changes of 15% QY decrease and 20% lifetime decrease that were selected based on the measured differences between AB0 and $AB_{sk}0$ (see SI Table S2), and (2) roughly match our experimental observations, as seen by comparing the results for low-FRET AB series clusters (for example, AB20) to their AB_{sk} counterparts ($AB_{sk}20$, which is ~23% dimmer in green brightness and ~13% shorter donor lifetime per Supplementary Table 3). This correlation supports the choice of simulation parameters.

Supplementary Note 6: Effects of salt and pH on FRETfluor properties

To investigate the sensitivity of FRETfluors to the surrounding solution environment, we measured the effects of varying pH and salt conditions on the measured spectroscopic parameters of a representative set of 10 FRETfluors. The FRETfluors were chosen to span the whole FRET range and included multiple members of each type of construct family.

We performed four different experiments using the same mixture of FRETfluors in different buffer conditions. For three experiments, the same buffer at three different pH (6.51, 7.4, 8.63) was used for the measurement (no salt added). For one experiment, we added 150 mM of NaCl to the neutral pH buffer to test the effects of salt.

To clearly visualize all the populations in the FRET versus donor lifetime projection, we first found the mean and the standard deviations of each tag in the data for all four experiments. In Supplementary Fig. 9, each tag's cluster location and spread are represented by an ellipse centered at its respective mean FRET and lifetime. The radius of the ellipse is twice the calculated standard deviation in each direction.

Our results show that there is no notable change in the FRETfluors signals within the pH range tested. However, at the higher salt concentration, the signal of the $AB_{in}6$ and $AB_{in}12$ is slightly shifted to lower donor lifetime. These tags contain an extra Cy3 outside of FRET range that is singly-tethered to the bridge strand, and therefore might be more influenced by the dielectric properties of the solution than the doubly-tethered Cy dyes. At the high salt concentration tested, many FRETfluors were also slightly dimmer. At high salt, the original, cap, and internal modified tags were each about 8-10% dimmer. The skip modified tags were only 2-4% dimmer. This further suggests that salt might be differentially influencing the photophysics of different types of FRETfluor constructs, indicating that FRETfluor clusters should be carefully calibrated across all environmental conditions to be used, and/or that sets of FRETfluors that are robustly mutually identifiable even in the presence of such shifts should be selected for use.

In the future, we envision selecting FRETfluor sets with clusters spaced far enough apart to allow for small shifts due to potential environment or target effects. If, as our current data suggests, construct families exhibit similar environmentally-induced shifts, these patterns could prove useful to maintain cluster separability even under changing conditions. Moreover, it may be the case that for certain applications, sensitivity of a FRETfluor signal to the target or environment will confer sensing functionality; for example, detecting a change in the target's composition, confirming attachment to a low molecular-weight target that does not change trapping characteristics, or providing additional information about the local environment composition.

Ultimately, the range of FRETfluor tuning that is possible with any particular dye chemistry will depend upon the mechanisms by which its photophysics can be tuned by the local construct structure and chemistry, balanced with the effects of the solution environment.

Supplementary Note 7: Identification of FRETfluors depends on # photons available

For this analysis, we parsed the data from each level into successive groups of *M* photons each (discarding remainder), with each group of *M* photons shown as one point on a scatter plot. We then measured the change in the standard deviation of the clusters for red brightness, green brightness, and green lifetime, as a function of the number of photons per point and calculated the effect this would have on misidentification of a typical FRETfluor, AB11, as compared to one or two close neighbors (AB10 and AB12) or as compared to more distal FRETfluor clusters (AB8, AB16).

Supplementary Note 8: Sequence of mRNAs and FRETfluor binding sites

The three mRNAs used in this work encode the enhanced Green Fluorescent Protein (EGFP; 996 nt), firefly luciferase (FLuc; 1929 nt), and ovalbumin (OVA; 1438 nt), which include proprietary tail sequences to help stabilize the mRNA against degradation. The sequences are given as follows:

EGFP mRNA:

```
[tail*]AUGGUGAGCAAGGGCGAGGAGCUGUUCACCGGGGUGGUGCCCAUCCUGGUCGAGCUGGACGGC
GACGUAAACGGCCACAAGUUCAGCGUGUCCGGCGAGGGCGAGGGCGAUGCCACCUACGGCAAGCUGACCC
UGAAGUUCAUCUGCACCACCGGCAAGCUGCCCGUGCCCUGGCCCACCCUCGUGACCACCCUGACCUACGG
CGUGCAGUGCUUCAGCCGCUACCCCGACCACAUGAAGCAGCACGACUUCUUCAAGUCCGCCAUGCCCGAA
GGCUACGUCCAGGAGCGCACCAUCUUCUUCAAGGACGACGGCAACUACAAGACCCGCGCCGAGGUGAAGU
UCGAGGGCGACACCCUGGUGAACCGCAUCGAGCUGAAGGGCAUCGACUUCAAGGAGGACGGCAACAUCCU
GGGGCACAAGCUGGAGUACAACUACAACAGCCACAACGUCUAUAUCAUGGCCGACAAGCAGAAGAACGGC
AUCAAGGUGAACUUCAAGAUCCGCCACAACAUCGAGGACGGCAGCGUGCAGCUCGCCGACCACUACCAGC
AGAACACCCCCAUCGGCGACGGCCCCGUGCUGCUGCCCGACAACCACUACCUGAGCACCCAGUCCGCCCU
GAGCAAAGACCCCAACGAGAAGCGCGAUCACAUGGUCCUGCUGGAGUUCGUGACCGCCGCCGGGAUCACU
CUCGGCAUGGACGAGCUGUACAAGUAA[tail*]
```

FLuc mRNA:

```
[tail*]AUGGAGGACGCCAAGAACAUCAAGAAGGGCCCCGCCCCCUUCUACCCCCUGGAGGACGGCACC
GCCGGCGAGCAGCUGCACAAGGCCAUGAAGCGGUACGCCCUGGUGCCCGGCACCAUCGCCUUCACCGACG
CCCACAUCGAGGUGGACAUCACCUACGCCGAGUACUUCGAGAUGAGCGUGCGGCUGGCCGAGGCCAUGAA
GCGGUACGGCCUGAACACCAACCACCGGAUCGUGGUGUGCAGCGAGAACAGCCUGCAGUUCUUCAUGCCC
GUGCUGGGCGCCCUGUUCAUCGGCGUGGCCGUGGCCCCCGCCAACGACAUCUACAACGAGCGGGAGCUGC
UGAACAGCAUGGGCAUCAGCCAGCCCACCGUGGUGUUCGUGAGCAAGAAGGGCCUGCAGAAGAUCCUGAA
CGUGCAGAAGAAGCUGCCCAUCAUCCAGAAGAUCAUCAUCAUGGACAGCAAGACCGACUACCAGGGCUUC
CAGAGCAUGUACACCUUCGUGACCAGCCACCUGCCCCCCGGCUUCAACGAGUACGACUUCGUGCCCGAGA
GCUUCGACCGGGACAAGACCAUCGCCCUGAUCAUGAACAGCAGCGGCAGCACCGGCCUGCCCAAGGGCGU
GGCCCUGCCCCACCGGACCGCCUGCGUGCGGUUCAGCCACGCCCGGGACCCCAUCUUCGGCAACCAGAUC
AUCCCCGACACCGCCAUCCUGAGCGUGGUGCCCUUCCACCACGGCUUCGGCAUGUUCACCACCCUGGGCU
ACCUGAUCUGCGGCUUCCGGGUGGUGCUGAUGUACCGGUUCGAGGAGGAGCUGUUCCUGCGGAGCCUGCA
GGACUACAAGAUCCAGAGCGCCCUGCUGGUGCCCACCCUGUUCAGCUUCUUCGCCAAGAGCACCCUGAUC
GACAAGUACGACCUGAGCAACCUGCACGAGAUCGCCAGCGGCGGCGCCCCCCUGAGCAAGGAGGUGGGCG
AGGCCGUGGCCAAGCGGUUCCACCUGCCCGGCAUCCGGCAGGGCUACGGCCUGACCGAGACCACCAGCGC
CAUCCUGAUCACCCCCGAGGGCGACGACAAGCCCGGCGCCGUGGGCAAGGUGGUGCCCUUCUUCGAGGCC
AAGGUGGUGGACCUGGACACCGGCAAGACCCUGGGCGUGAACCAGCGGGGCGAGCUGUGCGUGCGGGGCC
CCAUGAUCAUGAGCGGCUACGUGAACAACCCCGAGGCCACCAACGCCCUGAUCGACAAGGACGGCUGGCU
GCACAGCGGCGACAUCGCCUACUGGGACGAGGACGAGCACUUCUUCAUCGUGGACCGGCUGAAGAGCCUG
AUCAAGUACAAGGGCUACCAGGUGGCCCCCGCCGAGCUGGAGAGCAUCCUGCUGCAGCACCCCAACAUCU
UCGACGCCGGCGUGGCCGGCCUGCCCGACGACGACGCCGGCGAGCUGCCCGCCGCCGUGGUGGUGCUGGA
GCACGGCAAGACCAUGACCGAGAAGGAGAUCGUGGACUACGUGGCCAGCCAGGUGACCACCGCCAAGAAG
CUGCGGGGCGGCGUGGUGUUCGUGGACGAGGUGCCCAAGGGCCUGACCGGCAAGCUGGACGCCCGGAAGA
UCCGGGAGAUCCUGAUCAAGGCCAAGAAGGGCGGCAAGAUCGCCGUGUGA[tail*]
```

OVA mRNA:

[tail*]AUGGGCAGCAUCGGCGCCGCCAGCAUGGAGUUCUGCUUCGACGUGUUCAAGGAGCUGAAGGUG
CACCACGCCAACGAGAACAUCUUCUACUGCCCCAUCGCCAUCAUGAGCGCCCUGGCCAUGGUGUACCUGG
GCGCCAAGGACAGCACCCGGACCCAGAUCAACAAGGUGGUGCGGUUCGACAAGCUGCCCGGCUUCGGCGA
CAGCAUCGAGGCCCAGUGCGGCACCAGCGUGAACGUGCACAGCAGCCUGCGGGACAUCCUGAACCAGAUC
ACCAAGCCCAACGACGUGUACAGCUUCAGCCUGGCCAGCCGGCUGUACGCCGAGGAGCGGUACCCCAUCC
UGCCCGAGUACCUGCAGUGCGUGAAGGAGCUGUACCGGGGCGGCCUGGAGCCCAUCAACUUCCAGACCGC
CGCCGACCAGGCCCGGGAGCUGAUCAACAGCUGGGUGGAGAGCCAGACCAACGGCAUCAUCCGGAACGUG
CUGCAGCCCAGCAGCGUGGACAGCCAGACCGCCAUGGUGCUGGUGAACGCCAUCGUGUUCAAGGGCCUGU
GGGAGAAGACCUUCAAGGACGAGGACACCCAGGCCAUGCCCUUCCGGGUGACCGAGCAGGAGAGCAAGCC
CGUGCAGAUGAUGUACCAGAUCGGCCUGUUCCGGGUGGCCAGCAUGGCCAGCGAGAAGAUGAAGAUCCUG
GAGCUGCCCUUCGCCAGCGGCACCAUGAGCAUGCUGGUGCUGCUGCCCGACGAGGUGAGCGGCCUGGAGC
AGCUGGAGAGCAUCAUCAACUUCGAGAAGCUGACCGAGUGGACCAGCAGCAACGUGAUGGAGGAGCGGAA
GAUCAAGGUGUACCUGCCCCGGAUGAAGAUGGAGGAGAAGUACAACCUGACCAGCGUGCUGAUGGCCAUG
GGCAUCACCGACGUGUUCAGCAGCAGCGCCAACCUGAGCGGCAUCAGCAGCGCCGAGAGCCUGAAGAUCA
GCCAGGCCGUGCACGCCGCCCACGCCGAGAUCAACGAGGCCGGCCGGGAGGUGGUGGGCAGCGCCGAGGC
CGGCGUGGACGCCGCCAGCGUGAGCGAGGAGUUCCGGGCCGACCACCCCUUCCUGUUCUGCAUCAAGCAC
AUCGCCACCAACGCCGUGCUGUUCUUCGGCCGGUGCGUGAGCCCCUGA[tail*]

*Proprietary tail sequences (TriLink BioTechnologies)

The secondary structures of the mRNAs were predicted by RNAfold,[11,12] and the EGFP structure is shown in Supplementary Fig. 15 as an example. In each case, the target sequence is located in a high-confidence loop of the structure, highlighted here in gray and indicated for EGFP on the structure shown in Supplementary Fig. 15 by the location of the FRETfluor binding. All bridge sequences are given in Supplementary Table 1, with gray highlights indicating the complementary regions to the target mRNA sequences.

Supplementary Note 9: Labeling efficiency and measurement throughput tradeoffs

We did not attempt to optimize throughput in this work; the ABEL trap measures only one molecule at a time, and we did not limit the time spent on each molecule. For higher throughput, a statistically optimal number of photons necessary to correctly identify each species could be determined, and feedback could be turned off after that predetermined time for each detected event, with a small bias voltage to bring new sample into the sensing region. Parallelized microfluidic readout channels and spectroscopic detection could permit additional throughput gains. A related challenge will be quantitatively connecting levels and events measured to the stoichiometry of a mixture.

We show in this work that FRETfluors are particularly useful for wash-free sensing at ultra-low concentrations, but such conditions also necessarily present labeling efficiency challenges. When labeling at ultra-low concentrations, a high proportion of detected FRETfluors will be free rather than bound to a target molecule, lowering the rate of useful detected signals. It will be necessary to consider tradeoffs between labeling efficiency and throughput on an application-by-application basis. We used the exquisite sequence specificity of nucleic acid hybridization and the site-specificity of cysteine labeling on proteins to prevent cross-reactivity in the applications shown here, but we expect that other common targeting methods such as antibody recognition will bring their own optimization challenges in terms of efficiency, potential cross-reactivity, and label size.

Supplementary Note 10: Instrument correction parameters

FRET correction parameters: To obtain accurate FRET values that can be replicated on different optical setups, a few corrections need to be performed. These correction factors can be experimentally measured using Alternating Laser Excitation (ALEX).[13–15] The three main FRET corrections are the donor leakage α, normalization of effective quantum yields and detection efficiencies γ, and direct acceptor excitation by the green laser δ. Using these correction factors, the corrected FRET is given by equation 13 where $\mathrm{F_{A|D}}$ is the acceptor fluorescence upon donor excitation and similarly $\mathrm{F_{D|D}}$ is the donor fluorescence upon donor excitation and $\mathrm{F_{A|A}}$ is the acceptor fluorescence upon acceptor excitation.

$$E_{FRET} = \frac{F_{A|D} - \alpha F_{D|D} - \delta F_{A|A}}{\gamma F_{D|D} + F_{A|D} - \alpha F_{D|D} - \delta F_{A|A}} \qquad (13)$$

In the main figures for this paper, we show FRET values without performing any corrections (except for background subtraction). In this work, we are using FRET to create unique photophysical signatures and not to characterize a system or estimate distances using FRET. We have performed separate trapping experiments using ALEX on the same samples (data not shown). Using ALEX, we trapped a mixture containing 9 FRETfluors from the original design and determined the correction factors needed to arrive at accurate FRET values. Note that these ALEX experiments were performed using only one red and one green detection channel while the experiments from current work were done using two green and two red channels for anisotropy measurements. Regardless, these correction factors along with the g factor for our setup can be used to determine accurate FRET values for comparison across different instruments.

The correction parameters measured for our setup are: α = 0.08, δ = 0.05, and γ = 0.67.

Determination of the *g* factor: The g factor accounts for the difference in the detection efficiency of the detectors at the two polarizations. In our experiments, photons are first split by color, using a dichroic into red and green channels. Then, both the red and green channels are split using a polarizing beam splitter into parallel and perpendicular channels. Thus, we will have two g factors: one for the two green polarization channels (g_{green}) and one for the two red polarization channels (g_{red}). To experimentally determine the g factor, we use a concentrated sample of malachite green. First, a measurement is taken to record intensities of all four detectors using vertically polarized excitation (measurement #1). Then, the measurement is repeated using horizontally polarized excitation (measurement #2).

For simplicity, we use D_s and D_p to denote the detector that will accept s (vertical to the table) and p (horizontal to the table) polarized light as defined by the polarizing beam splitter. G factor is always used to scale the D_s detector. Vertically polarized excitation is parallel to the D_p detector and horizontally polarized excitation is parallel to the D_s detector. Assuming the sample polarization should be the same whether we are using the horizontally or vertically polarized excitation, we have the following relation where the left-hand side of the equation refers to the polarization determined using measurement #1 and right-hand side refers to measurement #2.

$$\frac{D_p^1 - gD_s^1}{D_p^1 - gD_s^1} = \frac{gD_s^2 - D_p^2}{gD_s^2 + D_p^2} \qquad (14)$$

Solving for g results in:

$$g = \sqrt{\frac{D_p^1 D_p^2}{D_s^1 D_s^2}} \quad (15)$$

Our measured g factors are: $g_{green} = 1.0481$ and $g_{red} = 1.2998$.

Supplementary References

1. Wong, E. D. *et al.* Saccharomyces genome database update: server architecture, pan-genome nomenclature, and external resources. *GENETICS* **224**, iyac191 (2023).
2. Engel, S. R. *et al.* New data and collaborations at the *Saccharomyces* Genome Database: updated reference genome, alleles, and the Alliance of Genome Resources. *Genetics* **220**, iyab224 (2022).
3. *Principles of Fluorescence Spectroscopy*. (Springer US, Boston, MA, 2006). doi:10.1007/978-0-387-46312-4.
4. Wang, Q. & Moerner, W. E. Dissecting pigment architecture of individual photosynthetic antenna complexes in solution. *Proc. Natl. Acad. Sci.* **112**, 13880–13885 (2015).
5. Squires, A. H. *et al.* Single-molecule trapping and spectroscopy reveals photophysical heterogeneity of phycobilisomes quenched by Orange Carotenoid Protein. *Nat. Commun.* **10**, 1–12 (2019).
6. Bates, A. D. & Maxwell, A. *DNA Topology*. (Oxford University Press, Oxford ; New York, 2005).
7. Mujumdar, R. B., Ernst, L. A., Mujumdar, S. R., Lewis, C. J. & Waggoner, A. S. Cyanine dye labeling reagents: Sulfoindocyanine succinimidyl esters. *Bioconjug. Chem.* **4**, 105–111 (1993).
8. Sanborn, M. E., Connolly, B. K., Gurunathan, K. & Levitus, M. Fluorescence Properties and Photophysics of the Sulfoindocyanine Cy3 Linked Covalently to DNA. *J. Phys. Chem. B* **111**, 11064–11074 (2007).
9. Lerner, E. *et al.* FRET-based dynamic structural biology: Challenges, perspectives and an appeal for open-science practices. *eLife* **10**, e60416 (2021).
10. Goldsmith, R. H. & Moerner, W. E. Watching conformational-and photodynamics of single fluorescent proteins in solution. *Nat. Chem.* **2**, 179–186 (2010).
11. Hofacker, I. L. Vienna RNA secondary structure server. *Nucleic Acids Res.* **31**, 3429–3431 (2003).
12. Lorenz, R. *et al.* ViennaRNA Package 2.0. *Algorithms Mol. Biol.* **6**, 26 (2011).
13. Kapanidis, A. N. *et al.* Alternating-Laser Excitation of Single Molecules. *Acc. Chem. Res.* **38**, 523–533 (2005).
14. Lee, N. K. *et al.* Accurate FRET Measurements within Single Diffusing Biomolecules Using Alternating-Laser Excitation. *Biophys. J.* **88**, 2939–2953 (2005).
15. Hellenkamp, B. *et al.* Precision and accuracy of single-molecule FRET measurements—a multi-laboratory benchmark study. *Nat. Methods* **15**, 669–676 (2018).
